# Application of 3-nitrooxypropanol and canola oil to mitigate enteric methane emissions of beef cattle results in distinctly different effects on the rumen microbial community

**DOI:** 10.1186/s42523-022-00179-8

**Published:** 2022-05-31

**Authors:** Robert J. Gruninger, Xiu Min Zhang, Megan L. Smith, Limin Kung, Diwakar Vyas, Sean M. McGinn, Maik Kindermann, Min Wang, Zhi Liang Tan, Karen A. Beauchemin

**Affiliations:** 1 Agriculture and Agri-Food Canada, Lethbridge Research and Development Centre, Lethbridge, AB T1J 4B1 Canada; 2 CAS Key Laboratory for Agro-Ecological Processes in Subtropical Region, National Engineering Laboratory for Pollution Control and Waste Utilization in Livestock and Poultry Production, South Central Experimental Station of Animal Nutrition and Feed Science in the Ministry of Agriculture, Hunan Provincial Engineering Research Center for Healthy Livestock and Poultry Production, Institute of Subtropical Agriculture, Chinese Academy of Sciences, Changsha, 410125 Hunan China; 3 University of Chinese Academy of Sciences (UCAS), Beijing, 100049 China; 4 Department of Animal and Food Sciences, University of Delaware, Newark, DE 19716 USA; 5 Department of Animal Sciences, Institute of Food and Agricultural Sciences, University of Florida, Gainesville, FL 32611 USA; 6 DSM Nutritional Products, Animal Nutrition and Health, CH-4002 Basel, Switzerland

## Abstract

**Background:**

The major greenhouse gas from ruminants is enteric methane (CH_4_) which in 2010, was estimated at 2.1 Gt of CO_2_ equivalent, accounting for 4.3% of global anthropogenic greenhouse gas emissions. There are extensive efforts being made around the world to develop CH_4_ mitigating inhibitors that specifically target rumen methanogens with the ultimate goal of reducing the environmental footprint of ruminant livestock production. This study examined the individual and combined effects of supplementing a high-forage diet (90% barley silage) fed to beef cattle with the investigational CH_4_ inhibitor 3-nitrooxypropanol (3-NOP) and canola oil (OIL) on the rumen microbial community in relation to enteric CH_4_ emissions and ruminal fermentation.

**Results:**

3-NOP and OIL individually reduced enteric CH_4_ yield (g/kg dry matter intake) by 28.2% and 24.0%, respectively, and the effects were additive when used in combination (51.3% reduction). 3-NOP increased H_2_ emissions 37-fold, while co-administering 3-NOP and OIL increased H_2_ in the rumen 20-fold relative to the control diet. The inclusion of 3-NOP or OIL significantly reduced the diversity of the rumen microbiome. 3-NOP resulted in targeted changes in the microbiome decreasing the relative abundance of *Methanobrevibacter* and increasing the relative abundance of *Bacteroidetes*. The inclusion of OIL resulted in substantial changes to the microbial community that were associated with changes in ruminal volatile fatty acid concentration and gas production. OIL significantly reduced the abundance of protozoa and fiber-degrading microbes in the rumen but it did not selectively alter the abundance of rumen methanogens.

**Conclusions:**

Our data provide a mechanistic understanding of CH_4_ inhibition by 3-NOP and OIL when offered alone and in combination to cattle fed a high forage diet. 3-NOP specifically targeted rumen methanogens and partly inhibited the hydrogenotrophic methanogenesis pathway, which increased H_2_ emissions and propionate molar proportion in rumen fluid. In contrast, OIL caused substantial changes in the rumen microbial community by indiscriminately altering the abundance of a range of rumen microbes, reducing the abundance of fibrolytic bacteria and protozoa, resulting in altered rumen fermentation. Importantly, our data suggest that co-administering CH_4_ inhibitors with distinct mechanisms of action can both enhance CH_4_ inhibition and provide alternative sinks to prevent excessive accumulation of ruminal H_2_.

**Supplementary Information:**

The online version contains supplementary material available at 10.1186/s42523-022-00179-8.

## Background

Ruminant production systems need to embrace the challenge of reducing greenhouse gas emissions (GHG) to align with the goals of the Paris Agreement that provides a pathway forward to limit temperature rise to well below 2 °C and potentially below 1.5 °C. The major GHG from ruminants is enteric methane (CH_4_), which in 2010, was estimated at 2.1 Gt of CO_2_ equivalent, accounting for 4.3% of global anthropogenic GHG emissions [1]. Enteric CH_4_ is a potent GHG with a 28-times greater global warming potential than carbon dioxide (CO_2_) when compared on an equivalent basis (CO_2_e) over 100-years. Enteric CH_4_ emissions also represent a loss of 2–12% of the gross energy consumed by ruminants [2]. Developing effective mitigation strategies to reduce enteric CH_4_ from ruminants therefore has the potential to increase production efficiency and reduce the environmental impact of ruminant livestock.

Dietary supplements and feed additives have been extensively investigated for their potential to decrease enteric CH_4_ emissions from ruminants [3, 4]. Of the mitigation options available, synthesized inhibitors have been found to be highly effective in inhibiting methanogenesis in the rumen when supplemented to the diet as feed additives [4]. However, many of these compounds (e.g., bromochloromethane, chloroform) are highly toxic, cause undesirable side-effects, or decrease methanogenesis only transiently. One exception is 3-nitrooxypropanol (3-NOP; DSM Nutritional Products, Basel, Switzerland), an investigational compound that has been shown to consistently decrease enteric CH_4_ emissions by 20 to 80% with no signs of animal toxicity [5–8]. Hristov et al. [9] reported a 25 to 32% decrease in CH_4_ production in lactating dairy cows that persisted over a 12-week study when 3-NOP was included in the ration at 40 to 80 mg/kg dry matter (DM). A dose response study identified a quadratic decrease in enteric CH_4_ emissions in dairy cattle with maximum reduction of CH_4_ yield (g/kg DM intake (DMI)) with 3-NOP included at 100 to 200 mg/kg DM [10]. In a study with growing beef cattle, a 37 to 42% decrease in enteric CH_4_ emissions occurred over 238-days when 3-NOP was supplemented at 125 to 200 mg/kg DM [11]. Similarly, Martinez-Fernandez et al. [6] reported that CH_4_ emissions were decreased by 38% in steers fed 3-NOP (338 mg/kg DM). Furthermore, studies that have examined the impact of 3-NOP on fiber digestion in the rumen or total-tract have reported no negative effects [6, 12, 13].

3-Nitrooxypropanol is a structural analogue of methyl coenzyme M and acts as a competitive inhibitor that selectively binds to methyl coenzyme M reductase (MCR) [14]. Binding of 3-NOP facilitates the oxidization of the catalytic nickel ion from Ni^+^ to Ni^2+^ temporarily inactivating the MCR enzyme and inhibiting the last step of methanogenesis [14]. In vitro assays with pure cultures of key rumen microbes revealed that 3-NOP very specifically inhibited the growth of rumen methanogens at low doses and had limited effects on the growth characteristics of the rumen bacteria tested [14]. There have been few studies that have examined the impact of 3-NOP on the composition of the rumen microbial community. Haisan et al. [15] used qPCR to show that 3-NOP significantly decreased 16S rRNA gene copy numbers for methanogens, and tended to decrease the total number of bacteria in rumen contents of dairy cows. Another study using next-generation sequencing found that 3-NOP decreased microbial alpha diversity in the rumen and resulted in a decrease in the relative abundance of hydrogenotrophic methanogens but had limited effects on rumen bacteria [16]. Most recently, it was shown that 3-NOP reduced the alpha diversity of the microbial community colonizing the surface of feed in the rumen of beef heifers and specifically reduced the abundance of methanogens without causing significant changes to the overall composition of the microbial community [13]. Other studies have reported that 3-NOP supplementation did not change the abundance of methanogens or total bacteria in beef cattle [17] or sheep [6]. Further research is needed to understand the effects that 3-NOP has on the composition and function of the rumen microbial community  when applied at different doses using various delivery methods to a range of diets. 

The addition of lipids to the diet of ruminants is another effective method of decreasing enteric CH_4_ emissions, and has the added benefit of enhancing digestible energy content of the feed [18]. The inclusion of lipids can also modify the fatty acid composition of milk and meat in a manner that enhances the health benefits associated with consuming these products [19]. The effectiveness with which lipid supplementation decreases enteric CH_4_ emissions varies and is dependent on diet composition, lipid composition (chain length and degree of saturation), inclusion level in the diet, and lipid source (oilseed vs. oil) [20]. In addition to reducing CH_4_ emissions, high fat diets are known to decrease fiber digestibility and may negatively affect DMI. Therefore, the maximum recommended total dietary lipid concentration is typically 60 to 80 g/kg DM (30 to 50 g/kg DM of added lipid) [20, 21]. Several meta-analyses of the literature found that 10 g/kg DM addition of lipid to the diet reduces CH_4_ yield by up to 5.6% [20, 22–24]. The microbial basis with which dietary lipids decrease methanogenesis is not fully understood but is thought to be due to: a decrease in the amount of organic matter fermented in the rumen; a decrease in the abundance of rumen methanogens and protozoa; and provision of alternative H_2_ sinks through biohydrogenation of unsaturated fatty acids [20]. Previous studies examining the impact of dietary lipids on the rumen microbial community did not observe a reduction in the abundance of rumen methanogens; however, the rumen microbial community was altered by specifically reducing the abundance of fibrolytic bacteria and protozoa [21, 25, 26].

The present study examines the impact of 3-NOP and canola oil supplementation alone, and in combination, on the rumen microbial community. An in depth analysis of the impacts of these treatments on rumen metabolism and enteric gas emissions has been published previously [27]. We examined the alterations in the rumen microbiome in relation to changes in enteric gas emissions and rumen fermentation in beef cattle fed a high forage diet. Rumen samples were collected over 12 h and the treatment effects were examined using a meta-taxonomic approach targeting the 16s rRNA gene. We hypothesized that 3-NOP has a highly targeted effect on rumen methanogens and that the inclusion of canola oil causes non-specific changes in the composition and function of the rumen microbial community. The aim of the study was to provide information that could be used to develop effective CH_4_ mitigation strategies.

## Results

### Sequencing of rumen samples

A total of 192 samples of rumen fluid and digesta were sequenced, resulting in 11,657,704 non-chimeric sequences after quality control and the identification of 6,217 unique ASVs from all samples. The minimum number of sequences per sample was 36,270 and the maximum was 95,904. Rarefaction analysis showed that sequencing depth was sufficient to capture most of the microbial diversity in the samples with rarefaction curves for all samples approaching an asymptote.

### Addition of 3-NOP and OIL reduced microbial diversity

The effect of 3-NOP, OIL and 3-NOP + OIL as a function of time on the alpha diversity of the rumen samples is shown in Table 1. Supplementing diets with 3-NOP resulted in a numerical reduction in observed ASVs at 0 h (*P* = 0.095), and a significant decrease in ASVs 6 and 12 h after feeding (*P* ≤ 0.05). 3-NOP decreased the phylogenetic diversity of rumen fluid at all of the sampling time points (*P* ≤ 0.05). The inclusion of OIL significantly decreased the number of ASVs and phylogenetic diversity of rumen fluid at all of the time points (*P* ≤ 0.001). No interactions between 3-NOP and OIL were found on the alpha diversity of rumen fluid. 3-NOP decreased alpha diversity of rumen digesta at 12 h (*P* ≤ 0.05) but there was no significant effect at 0 and 6 h (*P* > 0.05). The addition of OIL decreased the observed ASVs and phylogenetic diversity of rumen digest at all of the time points (*P* < 0.001). There was an interaction between 3-NOP and OIL on the phylogenetic diversity in rumen digesta at 0 h (*P* = 0.024) but not at 6 and 12 h.

**Table 1:** The impact of feeding diets supplemented with (+) and without (−) 3-NOP and OIL on the α-diversity of the rumen microbial community at 0, 6 and 12 h after feeding in beef cattle

Item	−3-NOP		+3-NOP		SEM	*P* value		
	−OIL	+OIL	−OIL	+OIL		3-NOP	OIL	3-NOP × OIL
**Rumen fluid**								
0 h								
Observed ASVs	560	406	501	383	23.7	0.095	< 0.001	0.46
Phylogenetic diversity	45.5	34.9	41.5	33.4	0.72	0.014	< 0.001	0.25
6 h								
Observed ASVs	536	379	457	335	28.8	0.021	< 0.001	0.49
Phylogenetic diversity	43.8	33.7	38.2	30.8	1.23	0.001	< 0.001	0.24
12 h								
Observed ASVs	528	393	429	344	32.1	0.002	< 0.001	0.27
Phylogenetic diversity	43.0	34.1	37.7	31.4	1.28	0.013	0.001	0.37
**Rumen Digesta**								
0 h								
Observed ASVs	820	614	738	657	32.3	0.55	< 0.001	0.062
Phylogenetic diversity	54.8^a^	42.6^c^	50.3^b^	43.6^c^	1.47	0.14	< 0.001	0.024
6 h								
Observed ASVs	747	592	696	603	17.7	0.46	< 0.001	0.26
Phylogenetic diversity	50.3	42.4	48.2	42.1	1.69	0.37	< 0.001	0.49
12 h								
Observed ASVs	810	604	686	582	7.1	0.017	< 0.001	0.084
Phylogenetic diversity	52.5	42.1	47.5	40.3	2.44	0.006	< 0.001	0.14

OIL, canola oil; 3-NOP, 3-nitrooxypropanol

^a,b,c^Values within a row with different letters differ (*P* ≤ 0.05)

### Effect of 3-NOP and OIL supplementation on microbial community composition

**Fig. 1:**
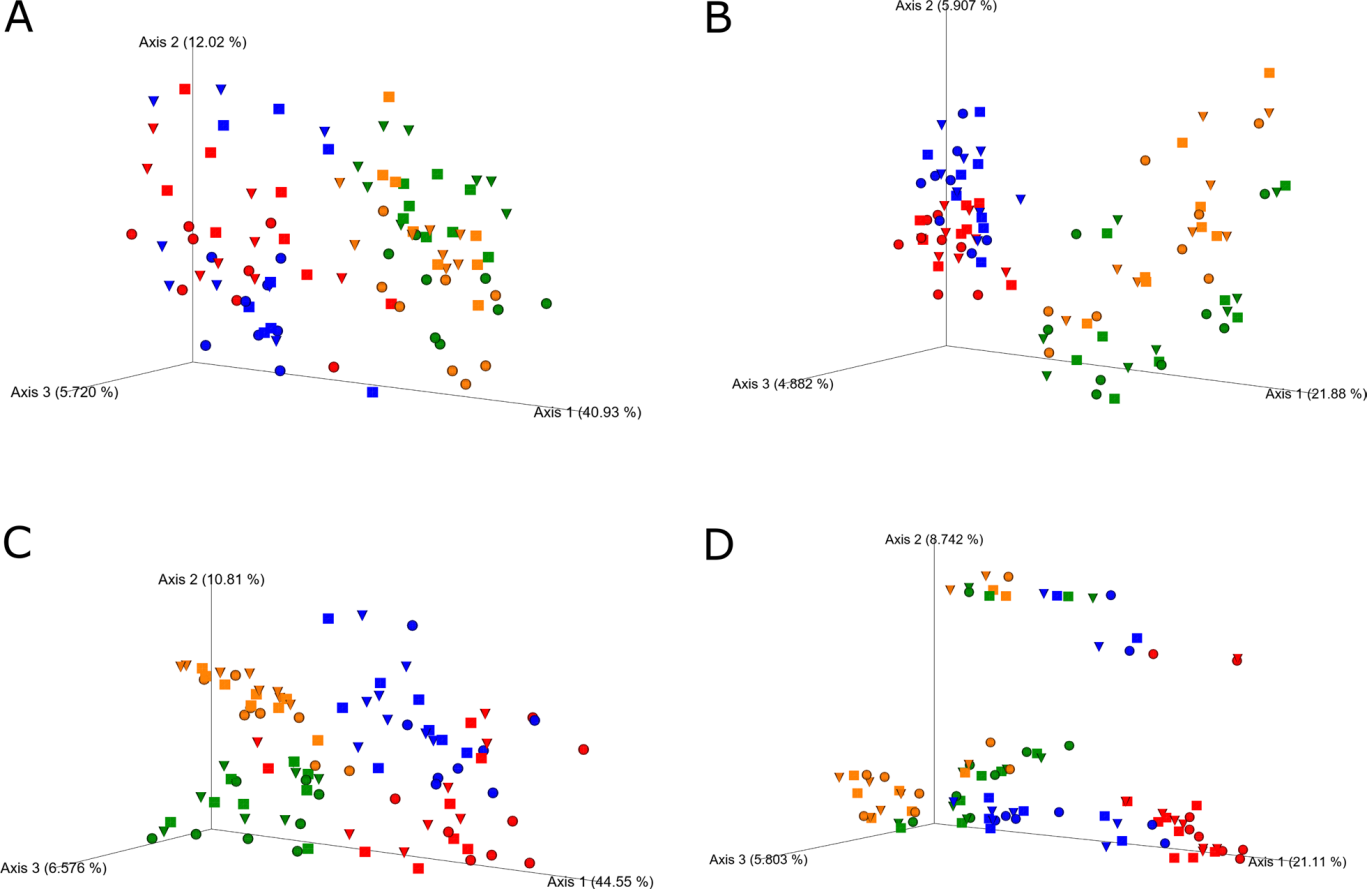
Impact of methane inhibitors on the bacterial and archaeal community in rumen digesta and rumen fluid from cattle fed a control diet (CON, red), a control diet supplemented with canola oil (OIL, green), 3-NOP (3-NOP, blue), or a combination of 3-NOP and canola oil (3-NOP + OIL, orange). Principle components plots are based on UniFrac distances and colored to show the impacts of time and treatment on microbiome composition. **A** weighted UniFrac plot of rumen fluid samples, **B** unweighted UniFrac plot of rumen fluid, **C** weighted UniFrac plot of rumen digesta, **D** unweighted UniFrac plot of rumen digesta. Samples taken prior to feeding are shown as circles, samples taken 6 h after feeding are shown as squares, samples taken 12 h after feeding are shown as triangles

Rumen fluid and rumen digesta samples clustered separately in PCoA plots based on both weighted and unweighted UniFrac distances (*P* < 0.001; Fig. 1). Samples also clustered separately by treatment in PcoA plots based on both weighted and unweighted UniFrac distances. For all plots, the control samples clustered distinctly from 3-NOP, OIL, or 3-NOP + OIL (*P* < 0.001) samples. The inclusion of OIL alone had the greatest effect on the composition of the microbial community with samples containing OIL significantly separated from control and 3-NOP samples (*P* < 0.001). Sampling time did not have a significant effect on the clustering of samples (*P* ≥ 0.26); however, a PERMANOVA analysis revealed that samples collected prior to morning feeding were different from 6- and 12-h samples (*P* = 0.006). There was no significant difference between 6 and 12 h samples (*P* = 0.56).

### Effect of 3-NOP and OIL on rumen methanogens

**Fig. 2:**
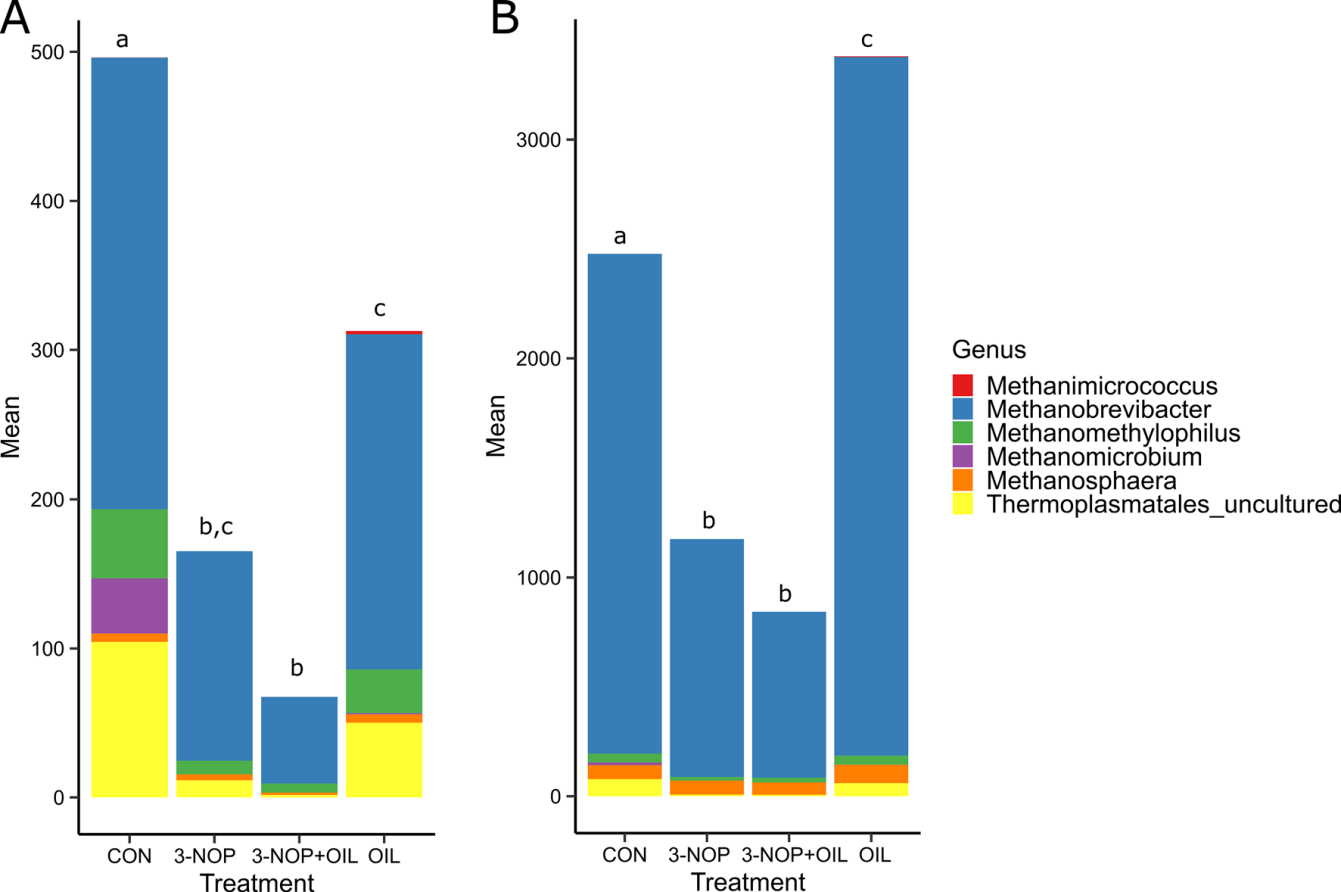
Impact of CH_4_ inhibitors on the abundance of rumen methanogens in **A** Rumen fluid and **B** Rumen digesta. The mean count of ASVs assigned to genera in the phylum *Euryarchaeota* is shown. Treatments resulting in statistically significant differences in the total count of ASVs classified within the phylum *Euryarchaeota* are indicted by a unique letter in each panel

The total number of ASVs that were assigned to the phylum *Euryarchaeota* in rumen digesta samples was significantly higher than in rumen fluid samples (Fig. 2). M*ethanobrevibacter* was the most abundant methanogen in both rumen solids and digesta. Sampling time did not significantly affect methanogen abundance in either rumen fluid or digesta (*P* ≥ 0.30); however, 3-NOP alone and in combination with OIL had a significant effect on methanogen abundance in both rumen fluid and digesta (*P* < 0.001). 3-NOP (*P* < 0.01) and 3-NOP + OIL (*P* < 0.001) significantly reduced the total number of *Euryarchaeota* ASVs in rumen fluid and rumen digesta samples (Fig. 2A, B respectively). The effects of 3-NOP and OIL on microbial abundance were generally independent of one another but there was a significant interaction between 3-NOP and OIL on the relative abundance of *Euryarchaeota* (*P* < 0.05, Tables 2 and 3). 3-NOP and 3-NOP + OIL caused significant decreases in the abundance of *Methanobrevibacter* (*P* < 0.05), *Methanomicrobium* (*P* < 0.001), *Methanomethylophilus* (*P* < 0.001), and an uncultured genus of *Thermoplasmatales* (*P* < 0.001). The addition of OIL decreased the abundance of *Euryarchaeota* in rumen fluid (*P* < 0.01). In contrast, the addition of OIL resulted in a significant increase in the abundance of *Euryarchaeota* in rumen digesta (*P* < 0.05). The effects observed for OIL treatment resulted in broad spectrum changes in the methanogen community and could not be attributed to a change in the abundance of a specific methanogen genus.

**Table 2:** Impact of feeding diets supplemented with (+) and without (−) 3-NOP and OIL on the relative abundance of phyla identified in rumen fluid in beef cattle

Item	-3-NOP		+ 3-NOP		SEM	*P* value		
	-OIL	+ OIL	-OIL	+ OIL		3-NOP	OIL	3-NOP × OIL
0 h								
*Euryarchaeota*	1.18	0.71	0.62	0.21	0.165	< 0.001	0.002	0.80
*Bacteroidetes*	54.9^b^	59.7^ab^	57.4^ab^	62.7^a^	2.75	0.11	0.007	0.023
*Firmicutes*	19.3	21.8	18.6	21.2	3.03	0.60	0.052	0.93
*Fibrobacteres*	10.6	0.26	10.2	0.14	1.16	0.81	< 0.001	0.89
*Actinobacteria*	0.40	0.15	0.54	0.14	0.158	0.67	0.038	0.62
*Proteobacteria*	4.18	12.0	3.48	12.0	1.55	0.81	< 0.001	0.84
*Spirochaetae*	1.97	2.36	3.48	1.89	0.532	0.31	0.24	0.061
*Verrucomicrobia*	4.10	1.24	3.65	0.82	0.586	0.30	< 0.001	0.97
Others (< 0.5%)	3.31	1.79	2.09	0.94	0.341	< 0.001	< 0.001	0.43
F: B	0.32	0.34	0.29	0.31	0.009	0.17	0.43	0.95
6 h								
*Euryarchaeota*	0.89^a^	0.54^b^	0.17^c^	0.11^c^	0.072	< 0.001	0.002	0.016
*Bacteroidetes*	45.0	49.5	53.1	49.9	2.77	0.042	0.75	0.059
*Firmicutes*	25.5	24.6	18.3	25.1	2.92	0.21	0.27	0.15
*Fibrobacteres*	7.68	0.06	6.04	0.02	1.018	0.39	< 0.001	0.42
*Actinobacteria*	2.15	0.22	0.98	0.39	0.553	0.34	0.022	0.20
*Proteobacteria*	12.5	22.1	15.2	23.1	3.79	0.58	0.016	0.79
*Spirochaetae*	2.36	1.25	3.66	0.81	0.538	0.43	0.001	0.12
*Verrucomicrobia*	2.22	0.66	1.64	0.27	0.237	0.019	< 0.001	0.64
Others (< 0.5%)	1.72	1.02	0.89	0.34	0.192	< 0.001	< 0.001	0.54
F: B	0.50	0.44	0.28	0.43	0.040	0.076	0.28	0.086
12 h								
*Euryarchaeota*	0.85	0.63	0.26	0.12	0.086	< 0.001	0.003	0.47
*Bacteroidetes*	50.8	49.3	52.0	55.6	2.24	0.070	0.60	0.20
*Firmicutes*	17.3	20.4	13.6	19.5	2.20	0.21	0.023	0.46
*Fibrobacteres*	11.7	0.16	11.3	0.06	1.37	0.87	< 0.001	0.93
*Actinobacteria*	0.86	0.28	0.63	0.32	0.252	0.66	0.053	0.55
*Proteobacteria*	11.2	25.8	15.4	22.3	3.06	0.92	0.002	0.21
*Spirochaetae*	2.40	1.31	3.75	1.19	0.564	0.21	0.001	0.14
*Verrucomicrobia*	2.61	0.83	1.96	0.44	0.286	0.041	< 0.001	0.59
Others (< 0.5%)	2.26	1.19	1.08	0.50	0.188	< 0.001	< 0.001	0.21
F: B	0.32	0.38	0.23	0.31	0.026	0.037	0.042	0.39

OIL, canola oil; 3-NOP, 3-nitrooxypropanol; F: B, *Firmicutes*: *Bacteroidetes*

^a,b,c^Values within a row with different letters differ (*P* ≤ 0.05)

**Table 3:** Impact of feeding diets supplemented with (+) and without (−) 3-NOP and OIL on the relative abundance of phyla identified in rumen digesta in beef cattle at 0, 6 and 12 h after feeding

Item	−3-NOP		+3-NOP		SEM	*P* value		
	−OIL	+OIL	−OIL	+OIL		3-NOP	OIL	3-NOP × OIL
0 h								
*Euryarchaeota*	3.43^b^	6.04^a^	2.18^b^	1.69^b^	0.631	< 0.001	0.031	0.003
*Bacteroidetes*	24.4	36.9	30.0	39.0	1.56	0.005	< 0.001	0.15
*Firmicutes*	48.4	51.3	45.0	52.7	1.55	0.49	0.001	0.096
*Fibrobacteres*	14.6	0.06	11.8	0.05	1.45	0.33	< 0.001	0.34
*Actinobacteria*	1.22	0.74	1.58	1.17	0.368	0.25	0.19	0.91
*Proteobacteria*	1.15	2.28	0.90	2.53	0.293	0.98	< 0.001	0.40
*Spirochaetae*	4.43	1.54	6.73	1.81	0.707	0.078	< 0.001	0.16
*Verrucomicrobia*	0.72	0.29	0.67	0.30	0.080	0.71	< 0.001	0.67
F: B	1.92	1.37	1.47	1.33	0.118	0.026	0.002	0.063
Others (< 0.5%)	1.59	0.85	1.14	0.74	0.171	0.11	0.002	0.32
6 h								
*Euryarchaeota*	3.24^a^	4.11^a^	1.39^b^	0.96^b^	0.466	< 0.001	0.44	0.030
*Bacteroidetes*	23.8	33.2	30.5	35.5	1.34	0.001	< 0.001	0.056
*Firmicutes*	52.1^a^	51.6^ab^	46.6^b^	51.9^a^	1.43	0.052	0.068	0.032
*Fibrobacteres*	9.97	0.05	9.27	0.03	1.18	0.75	< 0.001	0.77
*Actinobacteria*	4.00	0.83	2.62	1.49	1.17	0.74	0.061	0.36
*Proteobacteria*	2.69	8.23	3.95	8.31	1.122	0.52	< 0.001	0.58
*Spirochaetae*	2.47	1.11	4.46	1.14	0.525	0.065	< 0.001	0.073
*Verrucomicrobia*	0.53	0.23	0.51	0.19	0.067	0.62	< 0.001	0.90
F: B	2.17^b^	1.54^a^	1.52^a^	1.45^a^	0.145	0.002	0.004	0.020
Others (< 0.5%)	1.18	0.60	0.73	0.50	0.170	0.10	0.023	0.29
12 h								
*Euryarchaeota*	3.11^b^	4.51^a^	1.48^c^	0.84^c^	0.498	< 0.001	0.25	0.005
*Bacteroidetes*	26.7	35.9	32.7	39.2	1.20	< 0.001	< 0.001	0.22
*Firmicutes*	50.0	49.2	44.9	50.1	1.52	0.14	0.13	0.044
*Fibrobacteres*	9.38	0.09	8.18	0.06	1.052	0.55	< 0.001	0.57
*Actinobacteria*	2.73	0.69	2.17	1.28	0.848	0.98	0.052	0.43
*Proteobacteria*	2.90	7.51	4.06	6.71	0.959	0.85	0.001	0.31
*Spirochaetae*	2.96^b^	1.14^c^	5.18^a^	1.19^c^	0.531	0.041	< 0.001	0.051
*Verrucomicrobia*	0.56	0.28	0.51	0.20	0.069	0.27	< 0.001	0.76
F: B	1.85	1.35	1.35	1.27	0.115	0.005	0.004	0.064
Others (< 0.5%)	1.69	0.69	0.90	0.46	0.342	0.14	0.046	0.42

OIL, canola oil; 3-NOP, 3-nitrooxypropanol; F: B, *Firmicutes*: *Bacteroidetes*

^a,b,c^Values within a row with different letters differ (*P* ≤ 0.05)

### Effect of 3-NOP and OIL on the bacterial community of the rumen

The addition of 3-NOP, OIL and 3-NOP + OIL resulted in significant changes to the composition of the rumen bacterial community. The relative abundance of bacterial taxa in rumen fluid and digesta samples as a function of treatment and time are shown in Tables 2 and 3, respectively. The most abundant phyla in all samples were *Bacteroidetes* and *Firmicutes* regardless of time and treatment. Together these two phyla made up between 68 to 92% of the sequences identified. There was no effect of 3-NOP addition on the abundance of *Bacteroidetes* in rumen fluid samples before feeding (*P* = 0.11) but there was a significant increase 6 h after feeding (*P* = 0.042) and a tendency for greater abundance in samples 12 h after feeding (*P* = 0.070). The addition of 3-NOP significantly increased the abundance of *Bacteroidetes* in rumen digesta at all of the time points (*P* ≤ 0.05). This effect was primarily due to an increase in the relative abundance of *Prevotella*_1 (Additional file 1: Table S1 and S2). The addition of OIL to the diet also resulted in an increase in the abundance of *Bacteriodetes* in the rumen fluid before morning feeding (*P* = 0.007), but not 6 h (*P* = 0.75) or 12 h later (*P* = 0.60). The effect of OIL on rumen digesta samples was similar, with a significant increase in the abundance of *Bacteroidetes* at all of the time points due to an increase in the relative abundance of *Prevotella*_1 (*P* < 0.001). Conversely, there were decreases in the abundance of other genera including RC9 gut group (*P* ≤ 0.01), S24-7 (*P* ≤ 0.02), and RF16 (0 h and 6 h *P* ≤ 0.002). A significant interaction between treatments was observed in rumen fluid prior to feeding but not at other time points. There was no significant interaction between OIL and 3-NOP in rumen digesta.

The addition of 3-NOP did not significantly change the relative abundance of *Firmicutes* in rumen fluid at any of the time points (Table 2). However, at the genus level, 3-NOP significantly altered the abundance of several uncharacterized genera within this phylum (Additional file 1: Table S1). In contrast, the abundance of *Firmicutes* in rumen digesta was reduced at 6 h after feeding (*P* = 0.052) but not in the 0 h samples (*P* = 0.49) or 12 h after feeding (*P* = 0.13) (Table 3). OIL increased the abundance of *Firmicutes* before feeding (*P* = 0.052) and 12 h after feeding (*P* = 0.023) but not 6 h after feeding (*P* = 0.27) in rumen fluid samples (Table 2). There was a significant increase in the abundance of *Firmicutes* in the rumen digesta as a result of OIL addition before feeding (*P* = 0.001) and a tendency to be higher at 6 h after feeding (*P* = 0.068), but no significant difference at 12 h after feeding (*P* = 0.14) (Table 3). At the genus level, there were significant decreases in the abundance in *Christensenellaceae* R-7 group at all of the time points in rumen digesta but not in rumen fluid (Additional file 1: Tables S1 and S2). OIL supplementation resulted in an increase in the relative abundance of *Ruminococcus*_1 (*P* ≤ 0.001) and *Succinoclasticum* (*P* ≤ 0.001) (Additional file 1: Tables S1 and S2). The majority of other genera within *Firmicutes* that showed significant change in abundance due to the addition of OIL were unknown and/or uncultured taxa. There was a significant interaction between treatments on the relative abundance of *Firmicutes* in rumen digesta 6 h (*P* = 0.032) and 12 h (*P* = 0.044) after feed was consumed. All treatments resulted in a significant decrease in the *Firmicutes*:*Bacteroidetes* ratio in rumen digesta samples primarily due to the increase in the abundance of *Prevotella*_1 (Additional file 1: Tables S1 and S2).

The relative abundance of *Proteobacteria* in both rumen fluid and digesta was not affected by the addition of 3-NOP (Tables 2 and 3). In contrast, OIL supplementation resulted in a significant increase in *Proteobacteria* in both rumen fluid and digesta at all time points (*P* < 0.001). The impact of OIL on *Proteobacteria* was primarily due to increases in the abundance of *Ruminobacter* and an uncultured group of *Succinivibrionaceae* (Additional file 1: Tables S1 and S2). The addition of 3-NOP did not affect the abundance of *Fibrobacteres*, *Spirochaetae*, or *Verrucomicrobia* in any of the samples but the addition of OIL reduced the abundance of all three of these phyla. The most dramatic effect of OIL was observed for the genus *Fibrobacter*. The addition of OIL to the diet resulted in a marked decrease in the number of sequences attributable to *Fibrobacter* (41- to 243- fold decrease; *P* < 0.001) in both rumen fluid and digesta at all of the time points (Tables 2 and 3). There was also an interaction between OIL and 3-NOP that resulted in a larger decrease in the abundance of *Fibrobacter* (75- to 382-fold decrease; *P* < 0.001) in animals receiving 3-NOP + OIL. The relative abundance of *Spirochaetae* in the rumen digesta was decreased at all of the time points in animals receiving OIL. Similarly, OIL decreased the abundance of *Spirochaetae* in rumen fluid 6 and 12 h after feeding but not in samples taken prior to morning feeding. All of these sequences were attributed to the genus *Treponema*_2 (Additional file 1: Tables S1 and S2). The abundance of *Verrucomicrobia* was lowered by the addition of OIL relative to control at all of the time points in both rumen fluid and rumen digesta.

### Addition of OIL altered the protozoal community in rumen fluid

The impact that the addition of OIL and 3-NOP had on the composition of rumen protozoa was assessed by microscopic analysis of rumen fluid samples (Table 4). The addition of 3-NOP did not alter the total number of protozoa or the composition of the protozoal community at any time point, in contrast to the addition of OIL, which resulted in a decrease (*P* < 0.001) in the total number of protozoa at all of the time points. OIL altered the composition of the protozoal community significantly decreasing the abundance of *Dasytricha spp.*, *Entodinium spp.*, *Ostracodinium spp.*, and *Osphyoscolex spp.* in all samples and *Metadinium spp.* at 0 h and 12 h. No interaction effects between 3-NOP and OIL were observed for protozoa.

**Table 4:** Impact of feeding diets supplemented with (+) and without (−) 3-NOP and OIL on the protozoal populations (log10 cell/mL) in the rumen fluid of beef at 0, 6 and 12 h after feeding

Item	−3-NOP		+3-NOP		SEM	*P* value		
	−OIL	+OIL	−OIL	+OIL		3-NOP	OIL	3-NOP × OIL
0 h								
*Isotricha spp.*	0.21	ND	0.26	ND	0.200	0.87	0.15	0.87
*Dasytricha spp.*	0.49	ND	0.54	ND	0.392	0.92	0.016	0.92
*Entodiniomorphs*								
*Entodinium spp.*	5.60	3.81	5.56	4.28	0.290	0.34	< 0.001	0.27
*Ostracodinium spp.*	0.47	ND	0.75	ND	0.384	0.50	0.005	0.50
*Metadinium spp.*	0.95	ND	0.92	0.21	0.562	0.67	< 0.001	0.80
*Osphyoscolex spp.*	0.68	ND	0.52	ND	0.387	0.69	0.005	0.69
Total	5.61	3.81	5.58	4.28	0.287	0.34	< 0.001	0.27
6 h								
*Isotricha spp.*	0.23	ND	0.24	ND	0.197	0.97	0.15	0.97
*Dasytricha spp.*	0.49	ND	0.48	ND	0.368	0.98	0.016	0.98
*Entodiniomorphs*								
*Entodinium spp.*	5.51	4.00	5.49	4.42	0.218	0.21	< 0.001	0.17
*Ostracodinium spp.*	0.43	ND	0.68	ND	0.349	0.52	0.005	0.52
*Metadinium spp.*	0.21	ND	0.23	ND	0.185	0.95	0.15	0.95
*Osphyoscolex spp.*	0.89	ND	0.47	ND	0.403	0.28	0.001	0.28
Total	5.52	4.00	5.50	4.42	0.218	0.21	< 0.001	0.17
12 h								
*Isotricha spp.*	ND	ND	0.23	ND	0.113	0.32	0.32	0.32
*Dasytricha spp.*	0.24	ND	0.46	ND	0.199	0.59	0.082	0.59
*Entodiniomorphs*								
*Entodinium spp.*	5.49	3.83	5.47	4.21	0.256	0.48	< 0.001	0.43
*Ostracodinium spp.*	0.48	ND	0.45	ND	0.225	0.95	0.043	0.95
*Metadinium spp.*	0.62	ND	0.44	ND	0.225	0.70	0.022	0.70
*Osphyoscolex spp.*	0.45	ND	0.49	ND	0.228	0.92	0.043	0.92
Total	5.50	3.83	5.48	4.21	0.255	0.48	< 0.001	0.44

OIL, canola oil, 3-NOP, 3-nitrooxypropanol, ND, not detected

### CH_4_ and H_2_ emissions

**Fig. 3:**
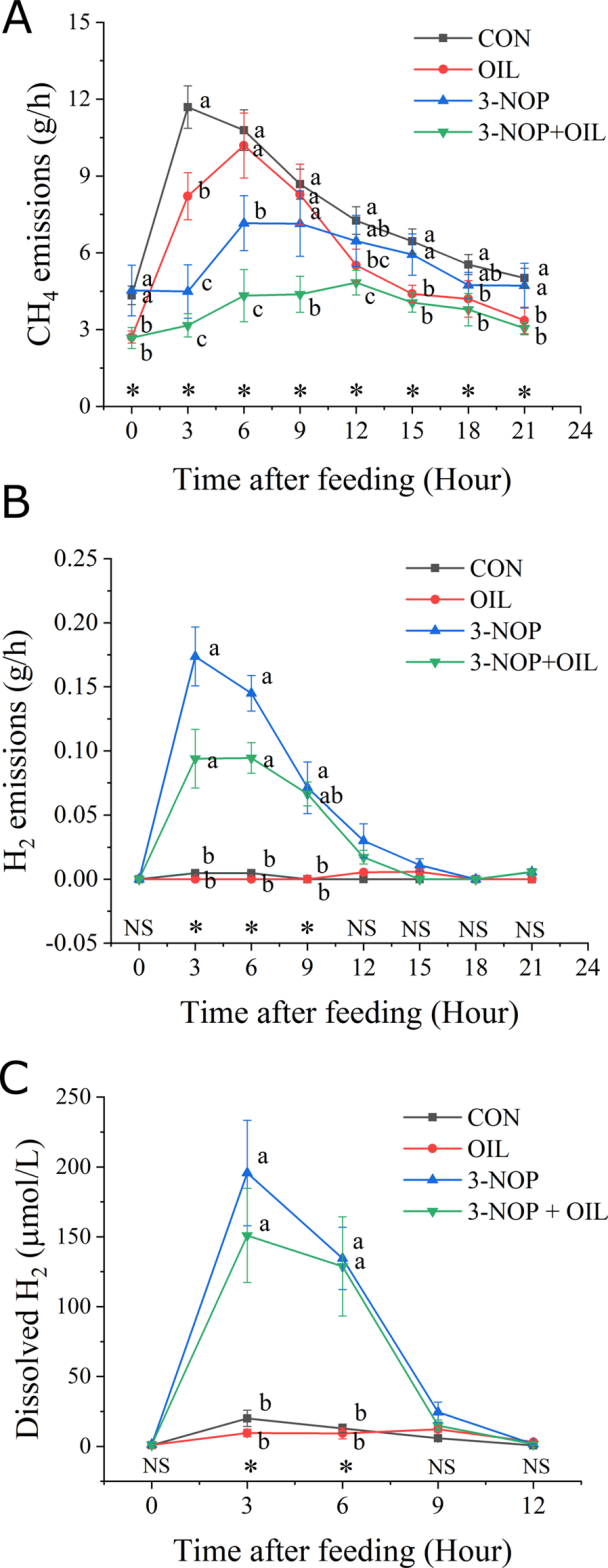
Enteric CH_4_ (**A**) and H_2_ (**B**) emissions, and dissolved H_2_ in rumen fluid for animals fed the CON, OIL, 3-NOP and 3-NOP + OIL dietary treatments over 24 h after feeding. Dissolved H_2_ levels were only measured over the first 12 h after feeding. Error bars indicate the SD. Asterisks (*) and different lowercase letters indicate time points in which the main effect of treatment is significant (*P* < 0.05), NS indicates no significant difference. CON = control, OIL = canola oil, 3-NOP = 3-nitrooxypropanol, 3-NOP + OIL = 3-nitrooxypropanol and canola oil

A detailed analysis of the treatment effects on daily gaseous emissions is presented by Zhang et al. [27]. The present study examined the impact of 3-NOP and OIL on CH_4_ and H_2_ emissions, and concentration of dH_2_, over the day. A typical diurnal pattern of CH_4_ emissions was observed for control cattle, with a rapid increase in CH_4_ emissions peaking at 11.7 g/h 3 h after feeding followed by a slow decrease to pre-feeding baseline levels of approximately 5 g/h (Fig. 3A). Compared to control diets, supplementation with 3-NOP or OIL decreased the rate of CH_4_ emission by 28.2% and 23.9%, respectively. There was a 51.4% decrease for the combined treatment which indicates that the effects of these mitigation strategies were additive (Fig. 3A). Feeding OIL or 3-NOP alone delayed the peak CH_4_ emission rate from 3 h in the control to 6 h after feeding, while emission rate for 3-NOP + OIL peaked 12 h after feeding. The greatest reduction in CH_4_ occurred in the first 6 h after feed consumption (Fig. 3A).

Enteric H_2_ emissions were minimal in cattle consuming the control or OIL supplemented diets (Fig. 3B). However, in cattle consuming 3-NOP supplemented diets, enteric H_2_ emissions rapidly increased 37-fold relative to control diets, peaking at 0.17 g/h, 3 h after feeding. Co-administering 3-NOP and OIL increased levels of H_2_ in the rumen 20-fold relative to control diets, peaking at 0.094 g/h 3–6 h after feeding. There was also an increase in dH_2_ concentration in rumen fluid (*P* < 0.05). The inclusion of 3-NOP resulted in a significant increase in the peak concentration of dH_2_ in rumen fluid (Fig. 3C). The peak concentration of dH_2_ was observed 3 h after feeding and reached a concentration of 20.1 µmol/L for the control diet. Inclusion of 3-NOP resulted in a 9.7-fold increase in the concentration of dH_2_ to 195.6 µmol/L compared with the control (*P* < 0.05). The addition of both 3-NOP and OIL resulted in an increase in dH_2_ concentration of 7.5-fold reaching 151.0 µmol/L compared with the control (*P* < 0.05).

### Changes in rumen fermentation and gas production were associated with shifts in the composition of the rumen microbiome

**Fig. 4:**
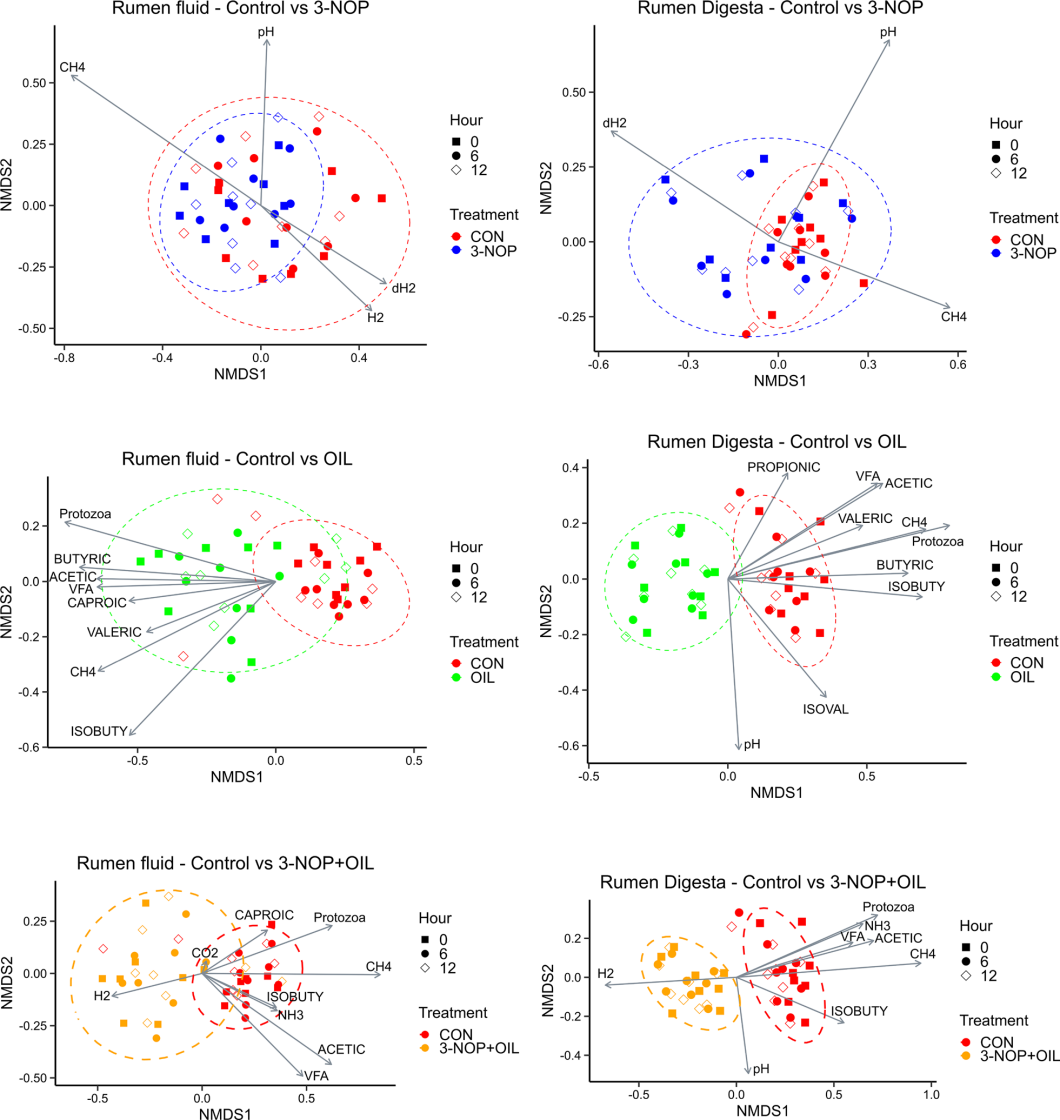
Impact of 3-NOP, OIL, and 3-NOP + OIL on the bacterial and archaeal community in rumen digesta and rumen fluid. Non-metric multidimensional scaling (NMDS) of the Bray–Curtis dissimilarity is shown to compare the samples as a function treatment and time. Vectors having a statistically significant association (*P* < 0.05) with the ordinations are included. Vector length is proportional to the degree of correlation between the environmental parameter and the ordination. Vector abbreviations: H_2_, CO_2_, CH_4_ emissions adjusted for dry matter intake. dH_2_: dissolved hydrogen, ISOBUTY: isobutyric acid, ISOVAL: isovaleric acid, VFA: total VFA concentration

Both CH_4_ mitigation strategies influenced a number of rumen fermentation parameters, gas production and dissolved H_2_ concentration. The influence of the treatments on animal metabolism is described in a separate manuscript [27]. Many of the observed changes in the rumen fermentation and gas production were significantly associated with the observed shifts in the rumen microbiome (Fig. 4). Samples from animals fed 3-NOP did not cluster separately in an NMDS ordination based on Bray–Curtis dissimilarity but pH (*R*^2^ = 0.67; *P* = 0.002) and g CH_4_/kg DMI (*R*^2^ = 0.38; *P* = 0.06) were significantly associated with the microbiome composition in rumen digesta. Rumen pH (*R*^2^ = 0.45; *P* = 0.034), acetate to propionate ratio (*R*^2^ = 0.45; *P* = 0.04), g H_2_/kg of DMI (*R*^2^ = 0.38; *P* = 0.06) and g CH_4_/kg DMI (*R*^2^ = 0.84; *P* < 0.0001) were significantly associated with the microbiome composition of rumen fluid. The inclusion of OIL resulted in substantial changes in the microbial community of rumen fluid and digesta and these shifts were significantly associated with total VFA concentration (*R*^2^ ≥ 0.48; *P* < 0.05), the molar proportions of acetate (*R*^2^ ≥ 0.47; *P* < 0.05), isobutyrate (*R*^2^ ≥ 0.53; *P* < 0.05), and butyrate (*R*^2^ ≥ 0.55; *P* < 0.01), total protozoa (*R*^2^ ≥ 0.45; *P* < 0.01), and g CH_4_/kg DMI (*R*^2^ 0.61; *P* < 0.01). In addition to these factors, pH (*R*^2^ ≥ 0.55; *P* ≤ 0.01) and valerate proportion (*R*^2^ ≥ 0.44; *P* ≤ 0.05) were associated with the observed changes in the microbial composition of rumen digesta due to OIL. The combination of NOP and OIL also resulted in distinct clustering of samples. Proportions of acetate (*R*^2^ ≥ 0.57; *P* < 0.01) and isobutyrate (*R*^2^ ≥ 0.42; *P* ≤ 0.05), total VFA (*R*^2^ ≥ 0.44; *P* ≤ 0.05), NH_3_ (*R*^2^ ≥ 0.45; *P* < 0.05), pH (*R*^2^ ≥ 0.47; *P* < 0.05), protozoa count (*R*^2^ ≥ 0.59; *P* < 0.01), g CH_4_/kg DMI (*R*^2^ 0.87; *P* < 0.001), and g H_2_/kg DMI (*R*^2^ 0.45; *P* < 0.05) were significantly associated with the changes in microbiome composition observed for the combined treatment.

## Discussion

We examined the impact of supplementing a high forage diet with the investigational CH_4_ inhibitor 3-NOP and high levels of lipid (canola oil; OIL), alone and in combination, on the composition of the rumen microbial community and the relationship of these changes to rumen fermentation and enteric gaseous emissions. We previously reported the CH_4_ emissions for the treatments used in the present study [27]; the cattle fed the diets that included 3-NOP (NOP and NOP + OIL) had 31.6% less CH_4_ yield (g/kg DMI) overall compared with those fed the diets that did not contain 3-NOP (control and OIL alone), while addition of OIL (OIL and NOP + OIL) decreased CH_4_ yield by 27.4% compared with diets that did not contain OIL (control and 3-NOP alone). The 31.6% decrease in CH_4_ yield with 3-NOP supplementation (200 mg/kg diet DM) that we observed is consistent with previous work that has reported a 30% to 40% decrease in CH_4_ yield in cattle fed a range of diets and doses of 3-NOP [7, 9–11, 28]. Similarly, the 27.4% decrease in CH_4_ yield for OIL was similar to expectations based on previous studies [20]. Importantly, co-administering 3-NOP and OIL treatments resulted in an incremental mitigation response (51% decrease compared with control). The present study indicates that although both CH_4_ mitigation approaches successfully decreased emissions, their impacts on the microbial community differed. Diets containing OIL caused substantial changes to the rumen microbial community, both in terms of the abundance of microbes and the microbes present, with these changes persisting over the course of the day. In comparison, 3-NOP targeted hydrogenotrophic methanogens with only minor effects on the bacterial community.

### 3-NOP reduced methanogenesis by reducing the abundance of hydrogenotrophic methanogens and shifted fermentation towards alternative H_2_ sinks

3-NOP (alone and in combination with OIL) decreased the bacterial alpha diversity as reflected by the decreased observed ASVs and phylogenetic diversity in rumen fluid samples but did not result in major changes in the composition of the rumen microbial community. The relative abundance of ASVs in *Bacteroidetes* was increased in 3-NOP and 3-NOP + OIL treatments resulting in a decrease in the *Firmicutes*:*Bacteroidetes* ratio. Rumen *Bacteroidetes* are net H_2_ utilizers [29], and the increased H_2_ levels associated with 3-NOP containing diets may have provided a niche for the proliferation of *Bacteroidetes*.

Our results provide strong support that the decrease in enteric CH_4_ that is observed in animals consuming 3-NOP results from the targeted mode of action on methanogenesis, causing a dramatic decrease in abundance of methanogens in rumen fluid and digesta. This observation is consistent with a previous in vitro study using pure cultures that showed 3-NOP had minimal impact on specific rumen bacteria, even though methanogen (*Methanothermobacter marburgensis*) growth and methanogenesis were inhibited [14]. 3-NOP is a structural analogue of methyl coenzyme M and acts as a competitive inhibitor that selectively binds to MCR and temporarily inactivates the enzyme by facilitating the oxidation of the catalytic nickel ion from Ni^+^ to Ni^2+^. The significant reduction in the relative abundance of *Euryarchaeota* observed in this study supports the previous results and shows that in vivo, 3-NOP also has high specificity for methanogens.

3-NOP supplementation resulted in changes in total VFA concentration in rumen fluid without substantial changes to the rumen microbial community. We hypothesize that the observed alterations in rumen fermentation are a result of alternative H_2_ sinks acting as terminal electron acceptors to capture some of the increased H_2_ observed in animals fed 3-NOP. There are a number of H_2_ sinks in the rumen, the most important of which is the conversion of CO_2_ to CH_4_. Methane formation is the main H_2_ sink in the rumen and a consequence of inhibiting methanogenesis by 3-NOP supplementation was an accumulation of dissolved H_2_ and gaseous H_2_ emissions. In the absence of methanogenesis, the formation of propionate, butyrate, and valerate can act as alternative H_2_ sinks [30]. Propionate is another principal alternative H_2_ sink in the rumen [31], and it appears that the accumulation of gaseous H_2_ and dissolved H_2_ in the rumen that resulted due to inhibiting methanogenesis shifted rumen metabolism in such a way that promoted propionate molar proportion, which may indicate a change in propionate production. It has also been suggested that when dissolved H_2_ concentration is elevated in the rumen, fermentation pathways that are net generators of H_2_ such as acetate production are unfavorable [32]. The observed increase in dissolved H_2_ concentration, accompanied by decreased molar proportions of acetate and increased proportion of propionate with 3-NOP (alone and in combination with OIL) supplementation observed in this study, are consistent with this observation [27]. Increased production of propionate does not incorporate all of the excess H_2_ when methanogenesis is inhibited and can lead to an increase in gaseous H_2_ emissions as we observed in the present study [12]. The increase in propionate molar percentage and decrease in acetate molar percentage that we observed with 3-NOP (alone and in combination with oil) is consistent with previous studies [6, 27, 33].

### Oil supplementation caused substantial changes in the composition of the rumen microbiome and altered rumen fermentation

OIL (alone and in combination with 3-NOP) had a substantial impact on the rumen microbial community and rumen fermentation. We observed reductions in the relative abundance of methanogens, significant changes in the composition of the rumen bacterial population, dramatic decreases in rumen protozoa and keystone fibrolytic bacteria, and changes in VFA concentration and proportions. The inclusion of OIL shifted rumen fermentation pathways and resulted in increased propionate and decreased acetate proportions. Lipids have been shown to decrease methanogenesis through various modes of action; they decrease the rumen fermentability of the organic matter when used to replace carbohydrates in the diet, they exert toxic effects on ruminal cellulolytic bacteria, protozoa, and methanogens, and the biohydrogenation of unsaturated fatty acids in the rumen acts as an alternative H_2_ sink [4]. Furthermore, lipid supplementation (≥ 4.0% of DMI) has previously been reported to increase propionate and decrease acetate percentages in beef and dairy cattle [23].

The dramatic decrease in protozoa numbers observed for the OIL treatments is consistent with expectations, as a protozoal defaunation is generally associated with a decrease in CH_4_ production [34]. While this relationship is not completely understood, it is thought to be related to the ability of protozoa to produce H_2_ and the close physical association between protozoa and methanogens resulting from the interspecies H_2_ transfer [34].

Although we did not directly measure ruminal fiber digestion we observed significant decreases in a number of important fiber degrading rumen microbes including *Fibrobacter*, *Bacteroidales* BS11, and *Bacteroidales* RF16 when OIL was included in the diet. *Fibrobacter* was decreased at all of the time points in both rumen fluid and rumen digesta by up to 382-fold. Eliminating this keystone member of the rumen microbial community would undoubtedly reduce the efficiency of cellulose degradation. An in vitro study examining the CH_4_ inhibition potential of Tucumã oil observed a 25% reduction in DM disappearance in the rumen, significantly reduced microbial richness, with a 16-fold reduction in the abundance of *Fibrobacter* but no specific effect on methanogens [25]. Interestingly, we also observed a significant increase in the abundance of *Ruminococcus*_1 in OIL samples. *Ruminococcus_*1 is known to contain the keystone fiber degrading bacteria *Ruminococcus flavifaciens* and *Ruminococcus albus* [35]. We speculate that the elimination of *Fibrobacter* from the rumen of these animals may have provided an opportunity for other fiber degrading bacteria that are less sensitive to fatty acids to occupy the niche previously filled by *Fibrobacter*. Several *Bacteroidetes* were also significantly less abundant and are also known to play important roles in carbohydrate metabolism. *Bacteroidales* BS11 gut group utilize hemicellulose monomeric sugars (e.g., xylose, fucose, mannose and rhamnose) and are involved in converting these to VFA [36]. *Bacteroidales* RF16 group are abundant in a range of ruminants when high forage diets are consumed suggesting a role in plant cell wall degradation [37–39]. The shift in VFA profile and the changes to the microbiome composition that accompanied OIL supplementation is consistent with a reduction in ruminal fiber degradation and suggests that fiber digestion was impaired in the animals consuming the OIL treatments [40]. This supposition is supported by the observed 18.0% and 23.3% decrease in total-tract digestibility of neutral detergent fiber when OIL was added to diets without and with 3-NOP, respectively [27].

A reduction in fiber digestion can favor a shift towards increased starch utilization in the rumen. For most forage diets, an increase in starch digestion would be of little consequence for animal metabolism because of the low dietary starch concentration. However, whole crop barley silage (950 g/kg DM) supplemented with barley grain was used in the present study resulting in a relatively high starch concentration of 261 g/kg DM. The observed increase in the abundance of the genera *Succinivibrio* and *Ruminobacter* within the phylum *Proteobacteria* in the OIL treatments are known to play a role in starch metabolism [41, 42]. *Succinivibriaceae* are known to metabolize starch and generate succinate which is an intermediate in the propionate pathway. *Succiniclasticum* was also significantly more abundant in OIL diets and can convert succinate into propionate [43]. This metabolic shift in the rumen would also contribute to the increased proportions of propionate and the low H_2_ emissions we observed in this treatment. Interestingly, several studies have found that *Succinivibrionaceae* is associated with reduced CH_4_ emissions, increased feed efficiency and higher propionate proportion [44, 45].

### 3-NOP and OIL have distinct mechanisms for reducing CH_4_ emissions

Combining mitigation strategies has the potential to further reduce enteric CH_4_ emissions from ruminant livestock and increase animal efficiency by increasing metabolizable energy availability. To date there has been little research conducted to identify effective combinations of inhibitors that have distinct modes of action and act synergistically or independently to reduce enteric CH_4_ production [4]. In the present study we observed very few interactions between 3-NOP and OIL, which is consistent with the unique mechanisms with which these compounds reduce enteric CH_4_ emissions. Notable exceptions where significant interactions were observed are: 1) acetate and propionate proportions, and 2) methanogen abundance. Compared to 3-NOP treatment alone, co-administering 3-NOP and OIL resulted in an increase in propionate proportion with a concomitant decrease in acetate proportion as well as alterations in the lipid composition of the rumen fluid [27]. While VFA molar proportion is not equivalent to VFA production, the change in proportions of individual VFA can be an indication of altered fermentation pathways in the rumen. The results suggest that with 3-NOP + OIL the excess H_2_ was consumed through increased propionate production and biohydrogenation, and consequently H_2_ emissions were less when 3-NOP and OIL were fed together compared with when 3-NOP was fed alone. The effect of 3-NOP and OIL on decreasing methanogen abundance is an important result and shows that combinations of CH_4_ inhibitors can result in substantial reductions in methanogens. One explanation for the interaction between these inhibitors is that the impact of lipid on the protozoal community effectively reduced the abundance of methanogens associated with rumen protozoa and 3-NOP reduced the abundance of free-living methanogens. Although the inclusion of both 3-NOP and OIL had incremental effects on enteric CH_4_ emissions, the high lipid content of these diets impacted the abundance of fiber degrading bacteria in the rumen and this may have had a negative effect on feed digestibility in these animals. For a high forage diet, the reduction in fiber degradation resulting from the inclusion of high concentrations of lipids may outweigh the CH_4_ mitigating effects these lipids have in high performing animals. Additional research is needed to identify optimal inclusion levels that will maximize CH_4_ mitigation without compromising animal performance.

## Conclusions

This study examined the impact of 3-NOP and canola oil supplementation as potential interventions to reduce enteric CH_4_ emissions from beef cattle. Our study found that these compounds are both effective treatments for reducing CH_4_ emissions; however, they have unique impacts on the rumen microbial community. 3-NOP is a highly targeted inhibitor that specifically reduces the activity of the dominant rumen methanogens and has minimal effects on ruminal bacteria and protozoa. In contrast, canola oil decreased the population of rumen protozoa and resulted in considerable changes to the bacterial community. There was a dramatic decrease in the abundance of key fiber degrading rumen microbes, in particular *Fibrobacter*. Importantly, co-administering CH_4_ inhibitors with distinct mechanisms of action can both enhance CH_4_ inhibition and provide alternative H_2_ sinks to prevent excessive accumulation of ruminal H_2_.

## Methods

The experiment was conducted at Agriculture and Agri-Food Canada’s Research and Development Centre in Lethbridge, AB, Canada. Animals were cared for in accordance with the guidelines of the Canadian Council on Animal Care (2009). Full details of the animal metabolism study are given in Zhang et al. [27].

### Experimental design and dietary treatments

Eight ruminally cannulated beef heifers (Angus cross, 732 ± 43 kg) were used in a double 4 × 4 Latin square design with four 28-d periods and 4 dietary treatments arranged as a 2 (3-NOP, with and without) × 2 (OIL, with and without) factorial. All animals were adapted to their respective diets from day 1–13 of each period and the remaining 15 days were used for measurements and sample collection. The dietary treatments were: 1) control (basal diet, **CON**); 2) 3-NOP alone (200 mg/kg of diet DM; **3-NOP,** DSM Nutritional Products Ltd., Kaiseraugst, Switzerland); 3) canola oil alone (50 g/kg DM, **OIL;** Loveland Industries, Inc., Loveland, CO, USA); and 4) 3-NOP (200 mg/kg of diet DM) and canola oil (50 g/kg DM) combined (**3-NOP + OIL**). Animals were blocked according to body weight and then randomly assigned to one of the 4 treatments. The animals were fed a high forage diet that consisted of (DM basis) 900 g/kg barley silage, 41.2 g/kg dry rolled barley grain, 50 g/kg supplement mix and 8.8 g/kg treatment mix (control or treatment). Both treatment mixes were prepared weekly and refrigerated, before being fully mixed with the diet. Total mixed rations were mixed daily and animals were fed at 10:00 h.

### Rumen sampling

Rumen content samples were obtained on day 14 of each period at 3 time points: prior to feeding (0 h), 6 h and 12 h after feeding. A representative 1 L sample of rumen contents (solid and liquid) was obtained from four different locations in the rumen (cranial, caudal, right and left sides of the rumen). The rumen content sample was filtered through 2 layers of polyester monofilament fabric (355 µm mesh opening) to separate the liquid and solid fractions. A 5 mL sample  of rumen fluid was obtained and preserved with methylgreen-formalin-saline solution, inverted ten times, and stored in the dark at room temperature (23 ± 2 °C) until protozoa were identified and counted as described previously [46]. Samples of filtered rumen fluid and samples of rumen digesta were snap frozen in liquid nitrogen and stored at -80 °C until DNA extraction.

### DNA extraction and 16s rRNA gene amplification

Frozen rumen samples were freeze dried and ground using a coffee grinder. Microbial DNA was extracted from ~ 0.1 g of the freeze dried, ground material using the Zymobiomics DNA extraction kit as per the manufacturer’s instructions (Zymo Research, Irvine CA). Concentration and purity of the extracted metagenomic DNA was determined by measuring the ratios of absorbance at 260/280 and 260/230 using a NanoDrop spectrophotometer (Thermo Fisher Scientific, Mississauga, ON, Canada).

Sequencing was performed at Genome Quebec Innovation Center (Montreal, Canada) using the Illumina MiSeq Reagent Kit v2 (500 cycle) following the manufacturer’s guidelines. The primers 515F (5′-GTGCCAGCMGCCGCGGTAA-3′) and 806R (5′-GGACTACHVGGGTWTCTAAT-3′) targeting the V4 region of the 16 s rRNA gene were used to examine both bacterial and archaeal diversity [47]. A 33 cycle PCR using 1 μL of a 1 in 10 dilution of genomic DNA and the Fast Start High Fidelity PCR System (Roche, Montreal, PQ) was conducted with the following conditions: 94 °C for 2 min, followed by 33 cycles of 94 °C for 30 s, 58 °C for 30 s, and 72 °C for 30 s, with a final elongation step at 72 °C for 7 min. Fluidigm Corporation (San Francisco, CA) barcodes were incorporated in a second PCR reaction using the FastStart High Fidelity PCR System under the following conditions: 95 °C for 10 min, followed by 15 cycles of 95 °C for 15 s, 60 °C for 30 s, and 72 °C for 1 min, followed by a final elongation step at 72 °C for 3 min. After amplification, PCR products were assessed in a 2% agarose gel to confirm adequate amplification. All samples were quantified using the Quant-iT PicoGreen dsDNA Assay Kit (Life Technologies, Carlsbad, CA) and were pooled in equal proportions. Pooled samples were then purified using calibrated Ampure XP beads (Beckman Coulter, Mississauga, ON). The pooled samples (library) were quantified using the Quant-iT PicoGreen dsDNA Assay Kit (Life Technologies, Carlsbad, CA) and the Kapa Illumina GA with Revised Primers-SYBR Fast Universal kit (Kapa Biosystems, Wilmington, MA). Average fragment size was determined using a LabChip GX (PerkinElmer, Waltham, MA, USA) instrument.

Raw fastq files were imported into Qiime2 for sequence analysis [48]. Primer and adapter sequences were removed from sequence files with the plugin ‘cutadapt’ [49]. Following removal of primer and adapter sequences, the program DADA2 [50] was used for quality control, filtering of any phiX reads present in the sequencing data, and removal of chimeric sequences. Amplicon Sequence variants (ASVs) at strain level resolution (> 99.9% id) were generated using DADA2 [50]. The Mafft program [51] was used to perform a multiple sequence alignment and to mask highly variable regions. A phylogenetic tree was generated with FastTree [52] and taxonomy was assigned to sequences using a Naïve-Bayes classifier trained with the Silva 128 reference database and the ‘feature-classifier’ plugin [53]. Sequences were subsampled to the lowest number of sequences found in all of the samples to ensure that α- and β-diversity analysis used the same number of sequences per sample. The plugin, ‘core-diversity-metrics’ was used to assess microbial diversity within (α-diversity) and between samples (β-diversity). The α-diversity indices: number of ASVs and Faith’s Phylogenetic Diversity were evaluated for treatment effects. β-Diversity analysis was carried out using weighted and unweighted UniFrac [54]. Environmental variables that had a significant impact on microbiome composition were identified in R using the envplot function. Sequences were deposited to the Small Reads Archive (NCBI) with accession number PRJNA680383.

### Enteric gas emissions and dissolved hydrogen

Enteric gas production (CH_4_ and H_2_) was measured in open-circuit calorimetry chambers from day 18 to 21 of every period according to the methods of Beauchemin and McGinn (2006) and McGinn et al. (2004) [24, 36]. All chambers were calibrated before and after the study by sequentially releasing 0, 0.2, and 0.4 L/min of CH_4_ into each chamber using a mass-flow meter (Omega Engineering, Stamford, CT). Slopes of the best fit calibration regressions were generated to correct emissions for each gas as detailed by McGinn et al. (2004) [55]. Variability in slopes across chambers was less than 5% and recovery rates ranged from 97 to 107%. Details of the chamber design and the calculation of CH_4_ emissions were reported by McGinn et al. [36]. Hydrogen (H_2_) production was measured at 30-min intervals every 3 h by collecting air samples from intake air ducts of each chamber. Hydrogen concentration was determined via a H_2_ breath tester (BreathTracker Digital Microlyzer, QuinTron Instrument Company, Inc., Milwaukee, WI, USA). Dissolved hydrogen (dH_2_) was measured in ruminal fluid samples collected on d 28 via the fistula at 0, 3, 6, 9 and 12 h after feeding. The dH_2_ concentration was measured using procedures described by Wang et al. [56]. Briefly, each ruminal fluid sample (35 mL) was quickly transferred into a 50-mL plastic syringe connected to a 20-mL syringe prefilled with 10 mL N_2_ gas. The N_2_ gas was then injected into the 50-mL syringe, and the syringe was vigorously shaken for 5 min to extract the gases dissolved in the ruminal fluid into the N_2_ gas phase. The extracted H_2_ concentration was determined by gas chromatography (Agilent 7890A, Agilent Inc., Palo Alto, CA, USA). The dH_2_ concentrations in the original ruminal fluid was calculated according to the equation given by Wang et al. (2016) [57].

### Calculations and statistical analysis

The daily CH_4_ flux was determined for each animal and expressed relative to DMI on the day of measurement (i.e., CH_4_ yield, g/kg DMI). Alpha diversity, relative abundance, protozoal counts (log_10_ transformed), rumen fermentation variables and gas data were analyzed by SAS (SAS Institute, Inc., 2015) using a mixed model procedure that included fixed effects of treatment (3-NOP, OIL and their interaction), and random effects of animal, period and group. The treatment effect was examined as a 2 × 2 factorial arrangement to determine the main effect of 3-NOP (control and OIL vs. 3-NOP and 3-NOP + OIL), OIL (control and 3-NOP vs. OIL and 3-NOP + OIL) and their interaction.

A PERMANOVA based statistical test was used to examine the influence of treatment, rumen phase (liquid vs. solid), or time of sampling on the composition of the microbial community. A Kruskal–Wallis test was used to examine the significance of treatment on Archaea. Independent pairwise comparisons with a Nemenyi test and a Chi-squared approximation was used to identify significant differences between treatments. *P* values were corrected for false discovery rates. Statistical significance was declared at *P* ≤ 0.05 and tendencies were declared at 0.05 < *P* ≤ 0.10.

## Supplementary Information

**Additional file 1: Table S1.** Temporal shifts in the relative abundance (≥ 0.5%) at genus level for rumen fluid. **Table S2.** Temporal shifts in the relative abundance (≥ 0.5%) at genus level for rumen digesta

## Data Availability

Sequence data has been submitted to the Sequence Read Archive with accession numbers PRJNA680383.
