# Supplementary material for: Application of 3-nitrooxypropanol and canola oil to mitigate enteric methane emissions of beef cattle results in distinctly different effects on the rumen microbial community
Source: Anim Microbiome. 2022 May 31;4:35. doi: 10.1186/s42523-022-00179-8 (PMC9158287; doi:10.1186/s42523-022-00179-8)
Supplement: Supplementary file 1 — Additional file 1: Table S1. Temporal shifts in the relative abundance (≥ 0.5%) at genus level for rumen fluid. Table S2. Temporal shifts in the relative abundance (≥ 0.5%) at genus level for rumen digesta [file 42523_2022_179_MOESM1_ESM.docx]

Supplementary Table 1: Temporal shifts in the relative abundance (≥0.5%) at genus level for rumen fluid

| Item | -3-NOP | |  | +3-NOP | | SEM | *P*-value | | |
| --- | --- | --- | --- | --- | --- | --- | --- | --- | --- |
|  | -OIL | +OIL |  | -OIL | +OIL |  | 3-NOP | OIL | 3-NOP × OIL |
| 0 h |  |  |  |  |  |  |  |  |  |
| *p_Euryarchaeota* |  |  |  |  |  |  |  |  |  |
| *g_Methanobrevibacter* | 0.90 | 0.58 |  | 0.54 | 0.18 | 0.146 | 0.009 | 0.016 | 0.88 |
| *p_Bacteroidetes* |  |  |  |  |  |  |  |  |  |
| *g_Provotella 1* | 26.8 | 41.9 |  | 33.5 | 45.9 | 2.15 | 0.013 | < 0.001 | 0.48 |
| *g_Rikenellaceae RC9 gut group* | 4.63 | 2.78 |  | 4.87 | 3.92 | 0.581 | 0.19 | 0.013 | 0.39 |
| *g_uncultured rumen bacterium_f_Bacteroidales S24-7 group* | 1.72 | 0.83 |  | 2.23 | 0.84 | 0.235 | 0.25 | < 0.001 | 0.26 |
| *g_Prevotellaceae NK3B31 group* | 0.68 | 2.83 |  | 0.92 | 1.20 | 0.530 | 0.17 | 0.022 | 0.07 |
| *g_uncultured rumen bacterium_f_Bacteroidales RF16 group* | 2.65 | 0.52 |  | 3.74 | 1.17 | 0.628 | 0.11 | 0.001 | 0.59 |
| *g_Prevotellaceae UCG-003* | 1.98 | 1.58 |  | 1.95 | 1.61 | 0.241 | 0.99 | 0.13 | 0.89 |
| *g_Prevotellaceae UCG-001* | 1.57 | 1.49 |  | 0.90 | 1.24 | 0.218 | 0.027 | 0.50 | 0.28 |
| *g_uncultured rumen bacterium_f_Bacteroidales BS11 gut group* | 4.14 | 1.34 |  | 1.87 | 1.19 | 0.981 | 0.21 | 0.08 | 0.28 |
| *g_uncultured rumen bacterium_f_Bacteroidales UCG-001* | 1.71 | 1.27 |  | 2.62 | 1.17 | 0.302 | 0.16 | 0.002 | 0.08 |
| *p_Firmicutes* |  |  |  |  |  |  |  |  |  |
| *g_Christensenellaceae R-7 group* | 2.97 | 3.11 |  | 2.40 | 2.65 | 0.493 | 0.22 | 0.64 | 0.89 |
| *g_unknown_f_Lachnospiraceae* | 1.12 | 2.77 |  | 1.29 | 2.56 | 0.358 | 0.94 | < 0.001 | 0.56 |
| *g_Ruminococcus 2* | 1.02 | 1.13 |  | 1.11 | 0.95 | 0.290 | 0.84 | 0.91 | 0.57 |
| *g_Succiniclasticum* | 1.32 | 1.76 |  | 1.56 | 1.74 | 0.192 | 0.55 | 0.11 | 0.49 |
| *g_Lachnospiraceae NK3A20 group* | 0.80 | 0.47 |  | 0.74 | 0.67 | 0.113 | 0.48 | 0.06 | 0.21 |
| *g_Ruminococcaceae NK4A214 group* | 1.04 | 0.83 |  | 0.75 | 0.65 | 0.118 | 0.027 | 0.14 | 0.62 |
| *g_Ruminococcus 1* | 0.60 | 0.86 |  | 0.47 | 0.99 | 0.122 | 0.97 | 0.001 | 0.19 |
| *g_[Eubacterium] coprostanoligenes group* | 0.64 | 0.75 |  | 0.54 | 0.92 | 0.104 | 0.70 | 0.012 | 0.14 |
| *g_Acetitomaculum* | 0.55 | 0.23 |  | 0.27 | 0.19 | 0.125 | 0.18 | 0.09 | 0.32 |
| *g_Saccharofermentans* | 0.61 | 0.83 |  | 0.59 | 0.87 | 0.181 | 0.94 | 0.054 | 0.81 |
| *g_uncultured_f_Ruminococcaceae* | 1.17 | 0.40 |  | 0.59 | 0.15 | 0.260 | 0.07 | 0.011 | 0.45 |
| *g_uncultured_f_Lachnospiraceae* | 0.43 | 0.47 |  | 0.28 | 0.26 | 0.081 | 0.018 | 0.94 | 0.67 |
| *g_Lachnospiraceae XPB1014 group* | 0.49 | 0.44 |  | 0.27 | 0.39 | 0.112 | 0.20 | 0.74 | 0.45 |
| *g_[Eubacterium] ruminantium group_f_ Lachnospiraceae* | 0.15 | 0.84 |  | 0.27 | 0.75 | 0.111 | 0.93 | < 0.001 | 0.30 |
| *g_unknown_f_ Ruminococcaceae* | 0.12 | 0.32 |  | 0.08 | 0.40 | 0.183 | 0.91 | 0.14 | 0.73 |
| *g_Clostridium sensu stricto 1* | 0.11 | 0.03 |  | 0.82 | 0.39 | 0.383 | 0.18 | 0.52 | 0.65 |
| *g_Lachnospiraceae AC2044 group* | 0.52 | 0.76 |  | 0.29 | 0.86 | 0.150 | 0.67 | 0.011 | 0.28 |
| *g_[Ruminococcus] gauvreauii group* | 0.19 | 0.38 |  | 0.14 | 0.22 | 0.047 | 0.011 | 0.001 | 0.14 |
| *g_Mogibacterium* | 0.11 | 0.14 |  | 0.08 | 0.15 | 0.028 | 0.68 | 0.049 | 0.48 |
| *g_Butyrivibrio 2* | 0.29 | 0.15 |  | 0.24 | 0.25 | 0.043 | 0.60 | 0.12 | 0.06 |
| *g_Moryella* | 0.09 | 0.17 |  | 0.15 | 0.17 | 0.026 | 0.30 | 0.06 | 0.16 |
| *p_Proteobacteria* |  |  |  |  |  |  |  |  |  |
| *g_Ruminobacter* | 1.64 | 7.74 |  | 1.34 | 7.97 | 1.57 | 0.98 | < 0.001 | 0.86 |
| *g_Succinivibrionaceae UCG-002* | 0.93 | 2.74 |  | 1.13 | 2.48 | 0495 | 0.96 | 0.003 | 0.63 |
| *p_Fibrobacteres* |  |  |  |  |  |  |  |  |  |
| *g_Fibrobacter* | 10.6 | 0.26 |  | 10.1 | 0.14 | 1.16 | 0.81 | < 0.001 | 0.89 |
| *p_Spirochaetae* |  |  |  |  |  |  |  |  |  |
| *g_Treponema 2* | 1.67 | 2.21 |  | 3.20 | 1.80 | 0.528 | 0.27 | 0.40 | 0.07 |
| *p_Verrucomicrobia* |  |  |  |  |  |  |  |  |  |
| *g_uncultured rumen bacterium_p_WCHB1-41* | 3.55 | 1.04 |  | 3.25 | 0.68 | 0.413 | 0.37 | < 0.001 | 0.93 |
| *p_Actinobacteria* |  |  |  |  |  |  |  |  |  |
| *g_Bifidobacterium* | 0.30 | 0.07 |  | 0.40 | 0.09 | 0.144 | 0.68 | 0.06 | 0.79 |
| 6 h |  |  |  |  |  |  |  |  |  |
| *p_Euryarchaeota* |  |  |  |  |  |  |  |  |  |
| *g_Methanobrevibacter* | 0.45 | 0.38 |  | 0.14 | 0.10 | 0.062 | < 0.001 | 0.32 | 0.78 |
| *p_Bacteroidetes* |  |  |  |  |  |  |  |  |  |
| *g_Provotella 1* | 24.3 | 36.3 |  | 32.0 | 38.2 | 2.42 | 0.039 | 0.001 | 0.19 |
| *g_Rikenellaceae RC9 gut group* | 3.79 | 2.20 |  | 3.98 | 2.57 | 0.374 | 0.42 | < 0.001 | 0.79 |
| *g_uncultured rumen bacterium_f_Bacteroidales S24-7 group* | 1.75 | 0.52 |  | 2.59 | 0.46 | 0.340 | 0.20 | < 0.001 | 0.15 |
| *g_Prevotellaceae NK3B31 group* | 0.39 | 2.24 |  | 0.84 | 1.06 | 0.436 | 0.36 | 0.013 | 0.046 |
| *g_uncultured rumen bacterium_f_Bacteroidales RF16 group* | 2.76 | 0.49 |  | 3.46 | 0.88 | 0.691 | 0.43 | 0.002 | 0.82 |
| *g_Prevotellaceae UCG-003* | 1.15 | 1.05 |  | 0.96 | 0.91 | 0.186 | 0.36 | 0.67 | 0.89 |
| *g_Prevotellaceae UCG-001* | 2.18 | 1.86 |  | 2.24 | 1.61 | 0.278 | 0.74 | 0.09 | 0.57 |
| *g_uncultured rumen bacterium_f_Bacteroidales BS11 gut group* | 2.04 | 0.78 |  | 1.42 | 0.33 | 0.485 | 0.26 | 0.018 | 0.85 |
| *g_uncultured rumen bacterium_f_Bacteroidales UCG-001* | 1.13 | 0.63 |  | 1.64 | 0.41 | 0.234 | 0.50 | 0.001 | 0.10 |
| *p_Firmicutes* |  |  |  |  |  |  |  |  |  |
| *g_Christensenellaceae R-7 group* | 1.96 | 2.16 |  | 1.33 | 1.23 | 0.265 | 0.002 | 0.82 | 0.49 |
| *g_unknown_f_Lachnospiraceae* | 1.80 | 3.83 |  | 1.70 | 1.93 | 0.542 | 0.05 | 0.031 | 0.08 |
| *g_Ruminococcus 2* | 3.21 | 2.17 |  | 2.08 | 6.25 | 1.351 | 0.28 | 0.25 | 0.06 |
| *g_Succiniclasticum* | 1.06 | 1.60 |  | 1.03 | 1.73 | 0.182 | 0.76 | 0.001 | 0.64 |
| *g_Lachnospiraceae NK3A20 group* | 1.48 | 0.89 |  | 0.93 | 0.82 | 0.195 | 0.09 | 0.06 | 0.18 |
| *g_Ruminococcaceae NK4A214 group* | 0.89 | 0.83 |  | 0.60 | 0.52 | 0.116 | 0.008 | 0.51 | 0.90 |
| *g_Ruminococcus 1* | 0.34 | 0.76 |  | 0.24 | 0.63 | 0.106 | 0.23 | < 0.001 | 0.89 |
| *g_[Eubacterium] coprostanoligenes group* | 0.37 | 0.86 |  | 0.25 | 0.99 | 0.094 | 0.98 | < 0.001 | 0.18 |
| *g_Acetitomaculum* | 1.44 | 0.27 |  | 0.38 | 0.31 | 0.363 | 0.15 | 0.09 | 0.13 |
| *g_Saccharofermentans* | 0.38 | 0.44 |  | 0.25 | 0.32 | 0.088 | 0.07 | 0.35 | 0.94 |
| *g_uncultured_f_Ruminococcaceae* | 3.60 | 0.85 |  | 0.85 | 0.43 | 0.613 | 0.009 | 0.008 | 0.043 |
| *g_uncultured_f_Lachnospiraceae* | 1.50 | 0.84 |  | 1.23 | 0.54 | 0.454 | 0.44 | 0.08 | 0.97 |
| *g_Lachnospiraceae XPB1014 group* | 0.41 | 0.49 |  | 0.23 | 0.32 | 0.057 | 0.002 | 0.11 | 0.84 |
| *g_[Eubacterium] ruminantium group_f_ Lachnospiraceae* | 0.10 | 0.56 |  | 0.07 | 0.37 | 0.057 | 0.06 | < 0.001 | 0.17 |
| *g_unknown_f_ Ruminococcaceae* | 0.23 | 1.11 |  | 0.07 | 1.37 | 0.654 | 0.94 | 0.09 | 0.74 |
| *g_Clostridium sensu stricto 1* | 1.29 | 0.26 |  | 2.41 | 1.92 | 1.06 | 0.20 | 0.47 | 0.80 |
| *g_Lachnospiraceae AC2044 group* | 0.40 | 0.57 |  | 0.18 | 0.40 | 0.103 | 0.07 | 0.06 | 0.85 |
| *g_[Ruminococcus] gauvreauii group* | 0.16 | 0.36 |  | 0.10 | 0.23 | 0.038 | 0.014 | < 0.001 | 0.41 |
| *g_Mogibacterium* | 0.12 | 0.16 |  | 0.08 | 0.11 | 0.025 | 0.06 | 0.13 | 0.96 |
| *g_Butyrivibrio 2* | 0.25 | 0.15 |  | 0.21 | 0.21 | 0.040 | 0.79 | 0.21 | 0.20 |
| *g_Moryella* | 0.22 | 0.28 |  | 0.27 | 0.31 | 0.049 | 0.45 | 0.29 | 0.83 |
| *p_Proteobacteria* |  |  |  |  |  |  |  |  |  |
| *g_Ruminobacter* | 7.96 | 16.5 |  | 11.1 | 17.0 | 3.54 | 0.57 | 0.030 | 0.67 |
| *g_Succinivibrionaceae UCG-002* | 2.55 | 3.60 |  | 2.31 | 3.46 | 0.840 | 0.82 | 0.20 | 0.95 |
| *p_Fibrobacteres* |  |  |  |  |  |  |  |  |  |
| *g_Fibrobacter* | 7.64 | 0.06 |  | 6.02 | 0.02 | 0.989 | 0.40 | < 0.001 | 0.42 |
| *p_Spirochaetae* |  |  |  |  |  |  |  |  |  |
| *g_Treponema 2* | 2.15 | 1.16 |  | 3.50 | 0.76 | 0.541 | 0.39 | 0.002 | 0.12 |
| *p_Verrucomicrobia* |  |  |  |  |  |  |  |  |  |
| *g_uncultured rumen bacterium_p_WCHB1-41* | 1.97 | 0.56 |  | 1.44 | 0.20 | 0.205 | 0.020 | < 0.001 | 0.62 |
| *p_Actinobacteria* |  |  |  |  |  |  |  |  |  |
| *g_Bifidobacterium* | 2.05 | 0.16 |  | 0.88 | 0.31 | 0.546 | 0.33 | 0.025 | 0.21 |
| 12 h |  |  |  |  |  |  |  |  |  |
| *p_Euryarchaeota* |  |  |  |  |  |  |  |  |  |
| *g_Methanobrevibacter* | 0.42 | 0.41 |  | 0.20 | 0.09 | 0.064 | < 0.001 | 0.28 | 0.43 |
| *p_Bacteroidetes* |  |  |  |  |  |  |  |  |  |
| *g_Provotella 1* | 26.8 | 31.4 |  | 28.4 | 40.7 | 1.97 | 0.002 | < 0.001 | 0.021 |
| *g_Rikenellaceae RC9 gut group* | 2.20 | 0.77 |  | 2.78 | 0.65 | 0.308 | 0.41 | < 0.001 | 0.22 |
| *g_uncultured rumen bacterium_f_Bacteroidales S24-7 group* | 0.54 | 4.49 |  | 0.96 | 1.13 | 0.908 | 0.08 | 0.019 | 0.030 |
| *g_Prevotellaceae NK3B31 group* | 3.93 | 0.96 |  | 5.39 | 2.13 | 0.936 | 0.14 | 0.002 | 0.87 |
| *g_uncultured rumen bacterium_f_Bacteroidales RF16 group* | 2.00 | 1.51 |  | 1.87 | 1.68 | 0.257 | 0.93 | 0.20 | 0.55 |
| *g_Prevotellaceae UCG-003* | 1.82 | 0.84 |  | 0.96 | 0.25 | 0.343 | 0.040 | 0.019 | 0.69 |
| *g_Prevotellaceae UCG-001* | 4.27 | 2.79 |  | 4.37 | 3.57 | 0.535 | 0.41 | 0.040 | 0.52 |
| *g_uncultured rumen bacterium_f_Bacteroidales BS11 gut group* | 1.46 | 1.14 |  | 0.83 | 0.94 | 0.202 | 0.043 | 0.58 | 0.28 |
| *g_uncultured rumen bacterium_f_Bacteroidales UCG-001* | 1.28 | 0.68 |  | 2.17 | 0.71 | 0.276 | 0.10 | 0.001 | 0.13 |
| *p_Firmicutes* |  |  |  |  |  |  |  |  |  |
| *g_Christensenellaceae R-7 group* | 1.67 | 1.87 |  | 1.23 | 1.11 | 0.207 | 0.003 | 0.85 | 0.37 |
| *g_unknown_f_Lachnospiraceae* | 1.23 | 3.23 |  | 1.22 | 1.47 | 0.465 | 0.038 | 0.011 | 0.040 |
| *g_Ruminococcus 2* | 1.56 | 1.62 |  | 1.15 | 2.75 | 0.527 | 0.47 | 0.11 | 0.13 |
| *g_Succiniclasticum* | 1.09 | 1.44 |  | 0.99 | 1.42 | 0.122 | 0.62 | 0.002 | 0.73 |
| *g_Lachnospiraceae NK3A20 group* | 0.79 | 0.57 |  | 0.67 | 0.66 | 0.118 | 0.89 | 0.32 | 0.36 |
| *g_Ruminococcaceae NK4A214 group* | 0.69 | 0.66 |  | 0.49 | 0.48 | 0.075 | 0.013 | 0.75 | 0.90 |
| *g_Ruminococcus 1* | 0.37 | 0.67 |  | 0.30 | 0.55 | 0.080 | 0.21 | 0.001 | 0.79 |
| *g_[Eubacterium] coprostanoligenes group* | 0.23 | 0.93 |  | 0.22 | 1.14 | 0.119 | 0.39 | < 0.001 | 0.32 |
| *g_Acetitomaculum* | 0.65 | 0.28 |  | 0.34 | 0.30 | 0.148 | 0.29 | 0.14 | 0.22 |
| *g_Saccharofermentans* | 0.45 | 0.49 |  | 0.25 | 0.30 | 0.090 | 0.017 | 0.60 | 0.93 |
| *g_uncultured_f_Ruminococcaceae* | 2.60 | 0.68 |  | 0.49 | 0.21 | 0.512 | 0.005 | 0.015 | 0.06 |
| *g_uncultured_f_Lachnospiraceae* | 0.58 | 0.63 |  | 0.38 | 0.30 | 0.151 | 0.07 | 0.91 | 0.69 |
| *g_Lachnospiraceae XPB1014 group* | 0.20 | 0.29 |  | 0.12 | 0.19 | 0.034 | 0.004 | 0.014 | 0.78 |
| *g_[Eubacterium] ruminantium group_f_ Lachnospiraceae* | 0.13 | 0.50 |  | 0.14 | 0.34 | 0.054 | 0.13 | < 0.001 | 0.12 |
| *g_unknown_f_ Ruminococcaceae* | 0.15 | 1.10 |  | 0.01 | 1.83 | 0.878 | 0.73 | 0.12 | 0.61 |
| *g_Clostridium sensu stricto 1* | 0.41 | 0.16 |  | 1.63 | 1.34 | 0.631 | 0.07 | 0.67 | 0.98 |
| *g_Lachnospiraceae AC2044 group* | 0.36 | 0.34 |  | 0.16 | 0.27 | 0.074 | 0.07 | 0.53 | 0.38 |
| *g_[Ruminococcus] gauvreauii group* | 0.10 | 0.27 |  | 0.10 | 0.16 | 0.031 | 0.07 | 0.001 | 0.07 |
| *g_Mogibacterium* | 0.10 | 0.12 |  | 0.07 | 0.09 | 0.018 | 0.15 | 0.26 | 0.96 |
| *g_Butyrivibrio 2* | 0.12 | 0.11 |  | 0.17 | 0.13 | 0.028 | 0.22 | 0.41 | 0.49 |
| *g_Moryella* | 0.14 | 0.20 |  | 0.14 | 0.19 | 0.034 | 0.88 | 0.09 | 0.96 |
| *p_Proteobacteria* |  |  |  |  |  |  |  |  |  |
| *g_Ruminobacter* | 4.98 | 16.2 |  | 9.44 | 12.4 | 2.94 | 0.90 | 0.023 | 0.17 |
| *g_Succinivibrionaceae UCG-002* | 4.06 | 6.16 |  | 3.61 | 5.98 | 1.080 | 0.77 | 0.044 | 0.90 |
| *p_Fibrobacteres* |  |  |  |  |  |  |  |  |  |
| *g_Fibrobacter* | 11.6 | 0.16 |  | 11.3 | 0.06 | 1.37 | 0.87 | < 0.001 | 0.93 |
| *p_Spirochaetae* |  |  |  |  |  |  |  |  |  |
| *g_Treponema 2* | 2.21 | 1.21 |  | 3.57 | 1.15 | 0.492 | 0.18 | 0.001 | 0.14 |
| *p_Verrucomicrobia* |  |  |  |  |  |  |  |  |  |
| *g_uncultured rumen bacterium_p_WCHB1-41* | 2.31 | 0.71 |  | 1.78 | 0.36 | 0.247 | 0.06 | < 0.001 | 0.69 |
| *p_Actinobacteria* |  |  |  |  |  |  |  |  |  |
| *g_Bifidobacterium* | 0.81 | 0.25 |  | 0.58 | 0.29 | 0.245 | 0.65 | 0.06 | 0.53 |

*p*: phylum; *f*: family; *g*: genus

OIL = canola oil, 3-NOP = 3-nitrooxypropanol.

Supplementary Table 2: Temporal shifts in the relative abundance (≥0.5%) at genus level for rumen digesta

| Item | -3-NOP | |  | +3-NOP | | SEM | *P*-value | | |
| --- | --- | --- | --- | --- | --- | --- | --- | --- | --- |
|  | -OIL | +OIL |  | -OIL | +OIL |  | 3-NOP | OIL | 3-NOP × OIL |
| 0 h |  |  |  |  |  |  |  |  |  |
| *p_Euryarchaeota* |  |  |  |  |  |  |  |  |  |
| *g_Methanobrevibacter* | 3.21 | 5.78 |  | 2.02 | 1.52 | 0.554 | < 0.001 | 0.031 | 0.003 |
| *p_Bacteroidetes* |  |  |  |  |  |  |  |  |  |
| *g_Provotella 1* | 11.8 | 25.9 |  | 15.7 | 26.8 | 1.38 | 0.05 | < 0.001 | 0.19 |
| *g_Rikenellaceae RC9 gut group* | 3.84 | 1.41 |  | 3.17 | 2.57 | 0.370 | 0.50 | < 0.001 | 0.016 |
| *g_uncultured rumen bacterium_f_Bacteroidales S24-7 group* | 1.79 | 2.55 |  | 3.58 | 1.51 | 0.528 | 0.43 | 0.17 | 0.006 |
| *g_Prevotellaceae NK3B31 group* | 1.63 | 1.33 |  | 1.75 | 1.85 | 0.488 | 0.51 | 0.83 | 0.69 |
| *g_uncultured rumen bacterium_f_Bacteroidales RF16 group* | 0.24 | 0.06 |  | 0.33 | 0.15 | 0.050 | 0.08 | 0.002 | 0.99 |
| *g_Prevotellaceae UCG-003* | 0.61 | 0.67 |  | 0.68 | 0.75 | 0.103 | 0.48 | 0.55 | 0.95 |
| *g_Prevotellaceae UCG-001* | 0.81 | 1.04 |  | 0.65 | 0.91 | 0.124 | 0.26 | 0.06 | 0.95 |
| *g_uncultured rumen bacterium_f_Bacteroidales BS11 gut group* | 0.40 | 0.66 |  | 0.50 | 0.45 | 0.128 | 0.58 | 0.32 | 0.13 |
| *g_uncultured rumen bacterium_f_Bacteroidales UCG-001* | 0.18 | 0.18 |  | 0.46 | 0.15 | 0.089 | 0.13 | 0.06 | 0.06 |
| *p_Firmicutes* |  |  |  |  |  |  |  |  |  |
| *g_Christensenellaceae R-7 group* | 8.92 | 6.31 |  | 7.69 | 6.34 | 0.753 | 0.42 | 0.012 | 0.39 |
| *g_unknown_f_Lachnospiraceae* | 4.56 | 4.92 |  | 5.27 | 5.53 | 0326 | 0.041 | 0.32 | 0.87 |
| *g_Ruminococcus 2* | 1.84 | 2.16 |  | 1.99 | 2.14 | 0.454 | 0.87 | 0.57 | 0.84 |
| *g_Succiniclasticum* | 2.35 | 4.07 |  | 2.79 | 3.39 | 0.253 | 0.64 | < 0.001 | 0.032 |
| *g_Lachnospiraceae NK3A20 group* | 2.69 | 3.65 |  | 3.28 | 3.18 | 0.306 | 0.85 | 0.17 | 0.09 |
| *g_Ruminococcaceae NK4A214 group* | 2.09 | 2.60 |  | 1.94 | 2.33 | 0.158 | 0.17 | 0.007 | 0.67 |
| *g_Ruminococcus 1* | 1.40 | 2.97 |  | 1.20 | 3.38 | 0.235 | 0.67 | < 0.001 | 0.20 |
| *g_[Eubacterium] coprostanoligenes group* | 0.87 | 2.08 |  | 0.69 | 2.69 | 0.184 | 0.23 | < 0.001 | 0.035 |
| *g_Acetitomaculum* | 1.62 | 1.22 |  | 0.90 | 1.22 | 0.249 | 0.13 | 0.86 | 0.13 |
| *g_Saccharofermentans* | 2.23 | 1.45 |  | 1.68 | 1.67 | 0.256 | 0.46 | 0.08 | 0.09 |
| *g_uncultured_f_Ruminococcaceae* | 0.88 | 0.58 |  | 0.70 | 0.29 | 0.253 | 0.33 | 0.14 | 0.81 |
| *g_uncultured_f_Lachnospiraceae* | 1.11 | 0.73 |  | 0.83 | 0.52 | 0.171 | 0.12 | 0.033 | 0.83 |
| *g_Lachnospiraceae XPB1014 group* | 1.76 | 1.10 |  | 1.50 | 1.13 | 0.160 | 0.38 | 0.001 | 0.25 |
| *g_[Eubacterium] ruminantium group_f_ Lachnospiraceae* | 0.55 | 1.79 |  | 0.60 | 1.57 | 0.173 | 0.61 | < 0.001 | 0.46 |
| *g_unknown_f_ Ruminococcaceae* | 0.37 | 0.86 |  | 0.23 | 0.76 | 0.410 | 0.77 | 0.21 | 0.97 |
| *g_Clostridium sensu stricto 1* | 0.04 | 0.02 |  | 0.15 | 0.28 | 0.107 | 0.10 | 0.60 | 0.48 |
| *g_Lachnospiraceae AC2044 group* | 1.10 | 0.97 |  | 0.92 | 1.07 | 0.175 | 0.85 | 0.95 | 0.40 |
| *g_[Ruminococcus] gauvreauii group* | 0.72 | 1.57 |  | 0.62 | 1.54 | 0.126 | 0.57 | < 0.001 | 0.77 |
| *g_Mogibacterium* | 0.73 | 1.26 |  | 0.66 | 1.15 | 0.066 | 0.17 | < 0.001 | 0.75 |
| *g_Butyrivibrio 2* | 1.59 | 0.37 |  | 1.09 | 0.50 | 0.112 | 0.11 | < 0.001 | 0.009 |
| *g_Moryella* | 0.36 | 0.87 |  | 0.57 | 1.13 | 0.069 | 0.002 | < 0.001 | 0.68 |
| *p_Proteobacteria* |  |  |  |  |  |  |  |  |  |
| *g_Ruminobacter* | 0.42 | 1.42 |  | 0.41 | 1.56 | 0.263 | 0.81 | < 0.001 | 0.77 |
| *g_Succinivibrionaceae UCG-002* | 0.18 | 0.44 |  | 0.17 | 0.43 | 0.077 | 0.92 | 0.001 | 0.98 |
| *p_Fibrobacteres* |  |  |  |  |  |  |  |  |  |
| *g_Fibrobacter* | 14.6 | 0.06 |  | 11.8 | 0.05 | 1.45 | 0.33 | < 0.001 | 0.34 |
| *p_Spirochaetae* |  |  |  |  |  |  |  |  |  |
| *g_Treponema 2* | 4.25 | 1.46 |  | 6.50 | 1.76 | 0.706 | 0.08 | < 0.001 | 0.18 |
| *p_Verrucomicrobia* |  |  |  |  |  |  |  |  |  |
| *g_uncultured rumen bacterium_p_WCHB1-41* | 0.46 | 0.19 |  | 0.47 | 0.20 | 0.051 | 0.77 | < 0.001 | 0.95 |
| *p_Actinobacteria* |  |  |  |  |  |  |  |  |  |
| *g_Bifidobacterium* | 0.66 | 0.17 |  | 0.99 | 0.63 | 0.345 | 0.24 | 0.20 | 0.84 |
| 6 h |  |  |  |  |  |  |  |  |  |
| *p_Euryarchaeota* |  |  |  |  |  |  |  |  |  |
| *g_Methanobrevibacter* | 2.97 | 3.85 |  | 1.28 | 0.87 | 0.401 | < 0.001 | 0.41 | 0.029 |
| *p_Bacteroidetes* |  |  |  |  |  |  |  |  |  |
| *g_Provotella 1* | 13.9 | 23.2 |  | 18.2 | 25.6 | 1.09 | 0.001 | < 0.001 | 0.31 |
| *g_Rikenellaceae RC9 gut group* | 2.75 | 0.90 |  | 2.55 | 1.60 | 0.279 | 0.36 | < 0.001 | 0.11 |
| *g_uncultured rumen bacterium_f_Bacteroidales S24-7 group* | 1.46 | 1.58 |  | 3.06 | 1.19 | 0.445 | 0.11 | 0.026 | 0.012 |
| *g_Prevotellaceae NK3B31 group* | 1.54 | 3.03 |  | 1.98 | 1.92 | 0.656 | 0.59 | 0.25 | 0.22 |
| *g_uncultured rumen bacterium_f_Bacteroidales RF16 group* | 0.31 | 0.07 |  | 0.41 | 0.13 | 0.064 | 0.21 | < 0.001 | 0.72 |
| *g_Prevotellaceae UCG-003* | 0.52 | 0.57 |  | 0.65 | 0.66 | 0.080 | 0.18 | 0.70 | 0.79 |
| *g_Prevotellaceae UCG-001* | 0.88 | 0.89 |  | 0.66 | 0.79 | 0.128 | 0.17 | 0.52 | 0.59 |
| *g_uncultured rumen bacterium_f_Bacteroidales BS11 gut group* | 0.23 | 0.25 |  | 0.30 | 0.15 | 0.057 | 0.67 | 0.12 | 0.05 |
| *g_uncultured rumen bacterium_f_Bacteroidales UCG-001* | 0.13 | 0.10 |  | 0.24 | 0.09 | 0.055 | 0.33 | 0.08 | 0.23 |
| *p_Firmicutes* |  |  |  |  |  |  |  |  |  |
| *g_Christensenellaceae R-7 group* | 7.62 | 5.58 |  | 6.16 | 4.54 | 0.617 | 0.05 | 0.006 | 0.74 |
| *g_unknown_f_Lachnospiraceae* | 5.00 | 5.40 |  | 6.19 | 4.77 | 0.488 | 0.57 | 0.30 | 0.07 |
| *g_Ruminococcus 2* | 4.26 | 3.98 |  | 3.56 | 5.96 | 0.883 | 0.44 | 0.21 | 0.11 |
| *g_Succiniclasticum* | 2.40 | 4.12 |  | 2.72 | 4.06 | 0.229 | 0.56 | < 0.001 | 0.41 |
| *g_Lachnospiraceae NK3A20 group* | 3.76 | 3.64 |  | 4.48 | 3.70 | 0.338 | 0.26 | 0.19 | 0.34 |
| *g_Ruminococcaceae NK4A214 group* | 1.94 | 2.07 |  | 1.61 | 1.68 | 0.105 | 0.001 | 0.28 | 0.79 |
| *g_Ruminococcus 1* | 1.17 | 2.41 |  | 0.87 | 2.03 | 0.203 | 0.09 | < 0.001 | 0.84 |
| *g_[Eubacterium] coprostanoligenes group* | 0.62 | 2.55 |  | 0.49 | 3.14 | 0.181 | 0.20 | < 0.001 | 0.05 |
| *g_Acetitomaculum* | 2.53 | 1.20 |  | 1.18 | 1.31 | 0.445 | 0.16 | 0.18 | 0.10 |
| *g_Saccharofermentans* | 1.48 | 0.92 |  | 0.98 | 0.85 | 0.160 | 0.031 | 0.012 | 0.11 |
| *g_uncultured_f_Ruminococcaceae* | 1.58 | 0.82 |  | 0.82 | 0.31 | 0.385 | 0.07 | 0.07 | 0.71 |
| *g_uncultured_f_Lachnospiraceae* | 2.27 | 1.09 |  | 1.09 | 0.68 | 0.593 | 0.11 | 0.11 | 0.42 |
| *g_Lachnospiraceae XPB1014 group* | 1.35 | 0.91 |  | 1.02 | 0.84 | 0.127 | 0.036 | 0.003 | 0.18 |
| *g_[Eubacterium] ruminantium group_f_ Lachnospiraceae* | 0.55 | 1.39 |  | 0.47 | 1.04 | 0.119 | 0.06 | < 0.001 | 0.21 |
| *g_unknown_f_ Ruminococcaceae* | 0.36 | 1.22 |  | 0.20 | 1.57 | 0.722 | 0.90 | 0.12 | 0.72 |
| *g_Clostridium sensu stricto 1* | 0.41 | 0.19 |  | 1.14 | 1.08 | 0.475 | 0.10 | 0.78 | 0.87 |
| *g_Lachnospiraceae AC2044 group* | 0.84 | 0.73 |  | 0.69 | 0.63 | 0.125 | 0.26 | 0.43 | 0.81 |
| *g_[Ruminococcus] gauvreauii group* | 0.78 | 1.39 |  | 0.71 | 1.48 | 0.117 | 0.94 | < 0.001 | 0.47 |
| *g_Mogibacterium* | 0.91 | 1.33 |  | 0.66 | 1.05 | 0.069 | < 0.001 | < 0.001 | 0.81 |
| *g_Butyrivibrio 2* | 1.47 | 0.36 |  | 1.31 | 0.49 | 0.126 | 0.87 | < 0.001 | 0.22 |
| *g_Moryella* | 0.58 | 1.03 |  | 0.81 | 1.23 | 0.068 | 0.004 | < 0.001 | 0.81 |
| *p_Proteobacteria* |  |  |  |  |  |  |  |  |  |
| *g_Ruminobacter* | 1.55 | 6.05 |  | 2.66 | 5.65 | 1.046 | 0.72 | 0.001 | 0.44 |
| *g_Succinivibrionaceae UCG-002* | 0.61 | 1.49 |  | 0.76 | 1.63 | 0.293 | 0.60 | 0.004 | 0.99 |
| *p_Fibrobacteres* |  |  |  |  |  |  |  |  |  |
| *g_Fibrobacter* | 9.94 | 0.05 |  | 9.23 | 0.03 | 1.174 | 0.75 | < 0.001 | 0.77 |
| *p_Spirochaetae* |  |  |  |  |  |  |  |  |  |
| *g_Treponema 2* | 2.38 | 1.07 |  | 4.35 | 1.12 | 0.522 | 0.06 | < 0.001 | 0.08 |
| *p_Verrucomicrobia* |  |  |  |  |  |  |  |  |  |
| *g_uncultured rumen bacterium_p_WCHB1-41* | 0.36 | 0.14 |  | 0.33 | 0.11 | 0.037 | 0.46 | < 0.001 | 0.99 |
| *p_Actinobacteria* |  |  |  |  |  |  |  |  |  |
| *g_Bifidobacterium* | 3.51 | 0.32 |  | 2.06 | 1.02 | 1.158 | 0.73 | 0.07 | 0.33 |
| 12 h |  |  |  |  |  |  |  |  |  |
| *p_Euryarchaeota* |  |  |  |  |  |  |  |  |  |
| *g_Methanobrevibacter* | 2.84 | 4.23 |  | 1.36 | 0.75 | 0.442 | < 0.001 | 0.24 | 0.006 |
| *p_Bacteroidetes* |  |  |  |  |  |  |  |  |  |
| *g_Provotella 1* | 16.6 | 25.5 |  | 20.2 | 28.9 | 0.95 | 0.001 | < 0.001 | 0.90 |
| *g_Rikenellaceae RC9 gut group* | 2.88 | 1.00 |  | 2.74 | 1.60 | 0.258 | 0.34 | < 0.001 | 0.13 |
| *g_uncultured rumen bacterium_f_Bacteroidales S24-7 group* | 1.32 | 1.41 |  | 2.63 | 1.40 | 0.473 | 0.16 | 0.22 | 0.15 |
| *g_Prevotellaceae NK3B31 group* | 1.25 | 3.00 |  | 1.81 | 2.12 | 0.604 | 0.78 | 0.08 | 0.21 |
| *g_uncultured rumen bacterium_f_Bacteroidales RF16 group* | 0.40 | 0.10 |  | 0.53 | 0.16 | 0.091 | 0.29 | 0.001 | 0.69 |
| *g_Prevotellaceae UCG-003* | 0.64 | 0.67 |  | 0.74 | 0.68 | 0.106 | 0.61 | 0.85 | 0.68 |
| *g_Prevotellaceae UCG-001* | 0.93 | 0.95 |  | 0.74 | 0.76 | 0.132 | 0.15 | 0.88 | 0.97 |
| *g_uncultured rumen bacterium_f_Bacteroidales BS11 gut group* | 0.26 | 0.36 |  | 0.30 | 0.13 | 0.066 | 0.08 | 0.51 | 0.017 |
| *g_uncultured rumen bacterium_f_Bacteroidales UCG-001* | 0.10 | 0.11 |  | 0.23 | 0.10 | 0.058 | 0.21 | 0.21 | 0.19 |
| *p_Firmicutes* |  |  |  |  |  |  |  |  |  |
| *g_Christensenellaceae R-7 group* | 7.88 | 6.22 |  | 6.07 | 4.66 | 0.623 | 0.011 | 0.019 | 0.84 |
| *g_unknown_f_Lachnospiraceae* | 4.79 | 4.88 |  | 5.70 | 4.86 | 0.356 | 0.22 | 0.30 | 0.20 |
| *g_Ruminococcus 2* | 3.33 | 2.98 |  | 3.47 | 5.32 | 0.802 | 0.11 | 0.33 | 0.16 |
| *g_Succiniclasticum* | 2.30 | 3.99 |  | 2.67 | 3.16 | 0.261 | 0.37 | < 0.001 | 0.026 |
| *g_Lachnospiraceae NK3A20 group* | 3.48 | 3.29 |  | 3.86 | 3.49 | 0.305 | 0.36 | 0.36 | 0.78 |
| *g_Ruminococcaceae NK4A214 group* | 2.02 | 2.03 |  | 1.74 | 1.58 | 0.116 | 0.002 | 0.47 | 0.44 |
| *g_Ruminococcus 1* | 1.49 | 2.58 |  | 1.25 | 2.39 | 0.225 | 0.31 | < 0.001 | 0.90 |
| *g_[Eubacterium] coprostanoligenes group* | 0.62 | 2.21 |  | 0.53 | 2.58 | 0.201 | 0.44 | < 0.001 | 0.21 |
| *g_Acetitomaculum* | 2.16 | 1.07 |  | 1.11 | 1.27 | 0.364 | 0.23 | 0.19 | 0.09 |
| *g_Saccharofermentans* | 1.72 | 1.05 |  | 1.04 | 0.90 | 0.184 | 0.005 | 0.006 | 0.06 |
| *g_uncultured_f_Ruminococcaceae* | 1.58 | 0.75 |  | 0.79 | 0.33 | 0.326 | 0.039 | 0.029 | 0.50 |
| *g_uncultured_f_Lachnospiraceae* | 1.32 | 0.95 |  | 1.01 | 0.65 | 0.318 | 0.28 | 0.19 | 0.98 |
| *g_Lachnospiraceae XPB1014 group* | 1.58 | 0.87 |  | 1.02 | 0.83 | 0.122 | 0.003 | < 0.001 | 0.008 |
| *g_[Eubacterium] ruminantium group_f_ Lachnospiraceae* | 0.77 | 1.63 |  | 0.67 | 1.30 | 0.134 | 0.049 | < 0.001 | 0.27 |
| *g_unknown_f_ Ruminococcaceae* | 0.31 | 1.40 |  | 0.20 | 2.07 | 0.911 | 0.75 | 0.11 | 0.66 |
| *g_Clostridium sensu stricto 1* | 0.22 | 0.10 |  | 0.72 | 0.71 | 0.324 | 0.10 | 0.86 | 0.87 |
| *g_Lachnospiraceae AC2044 group* | 1.05 | 0.77 |  | 0.73 | 0.80 | 0.161 | 0.30 | 0.46 | 0.19 |
| *g_[Ruminococcus] gauvreauii group* | 0.73 | 1.40 |  | 0.63 | 1.40 | 0.132 | 0.63 | < 0.001 | 0.64 |
| *g_Mogibacterium* | 0.81 | 1.22 |  | 0.68 | 0.97 | 0.062 | 0.004 | < 0.001 | 0.35 |
| *g_Butyrivibrio 2* | 1.17 | 0.34 |  | 0.95 | 0.47 | 0.093 | 0.63 | < 0.001 | 0.07 |
| *g_Moryella* | 0.49 | 0.87 |  | 0.65 | 1.09 | 0.069 | 0.011 | < 0.001 | 0.65 |
| *p_Proteobacteria* |  |  |  |  |  |  |  |  |  |
| *g_Ruminobacter* | 1.57 | 5.38 |  | 2.85 | 4.75 | 0.955 | 0.74 | 0.007 | 0.32 |
| *g_Succinivibrionaceae UCG-002* | 0.82 | 1.36 |  | 0.76 | 1.13 | 0.230 | 0.53 | 0.05 | 0.71 |
| *p_Fibrobacteres* |  |  |  |  |  |  |  |  |  |
| *g_Fibrobacter* | 9.36 | 0.09 |  | 8.15 | 0.06 | 1.051 | 0.55 | < 0.001 | 0.57 |
| *p_Spirochaetae* |  |  |  |  |  |  |  |  |  |
| *g_Treponema 2* | 2.85 | 1.09 |  | 5.06 | 1.16 | 0.530 | 0.041 | < 0.001 | 0.06 |
| *p_Verrucomicrobia* |  |  |  |  |  |  |  |  |  |
| *g_uncultured rumen bacterium_p_WCHB1-41* | 0.37 | 0.19 |  | 0.37 | 0.14 | 0.044 | 0.51 | < 0.001 | 0.51 |
| *p_Actinobacteria* |  |  |  |  |  |  |  |  |  |
| *g_Bifidobacterium* | 2.30 | 0.27 |  | 1.72 | 0.91 | 0.832 | 0.97 | 0.06 | 0.41 |

*p*: phylum; *f*: family; *g*: genus

OIL = canola oil, 3-NOP = 3-nitrooxypropanol.
